# Fingerprinting of neurotoxic compounds using a mouse embryonic stem cell dual luminescence reporter assay

**DOI:** 10.1007/s00204-016-1690-2

**Published:** 2016-03-25

**Authors:** Marilena Colaianna, Sten Ilmjärv, Hedi Peterson, Ilse Kern, Stephanie Julien, Mathurin Baquié, Giorgia Pallocca, Sieto Bosgra, Agapios Sachinidis, Jan G. Hengstler, Marcel Leist, Karl-Heinz Krause

**Affiliations:** 1 Department of Pathology and Immunology, Medical School, University of Geneva, Geneva, Switzerland; 2 Quretec Ltd, Ülikooli 6a, Tartu, Estonia; 3 Department of Pediatrics, Geneva University Hospital, Geneva, Switzerland; 4 Department of Genetic and Laboratory Medicine, Geneva University Hospital, Centre Medical Universitaire, Rue Michel-Servet, 1211 Geneva 4, Switzerland; 5 NEURIX SA, Geneva, Switzerland; 6 Doerenkamp-Zbinden Chair for In Vitro Toxicology and Biomedicine, University of Konstanz, Constance, Germany; 7 TNO, Zeist, The Netherlands; 8 Institute of Neurophysiology and Center for Molecular Medicine Cologne (CMMC), University of Cologne, Cologne, Germany; 9 Leibniz Research Centre for Working Environment and Human Factors (IfADo), Technical University of Dortmund, Dortmund, Germany; 10 BioMarin Pharmaceutical Inc., Leiden, The Netherlands

**Keywords:** Mouse embryonic stem cells, Neuroactivity, Neurotoxicity, In vitro screening, Neuronal differentiation

## Abstract

Identification of neurotoxic drugs and environmental chemicals is an important challenge. However, only few tools to address this topic are available. The aim of this study was to develop a neurotoxicity/developmental neurotoxicity (DNT) test system, using the pluripotent mouse embryonic stem cell line CGR8 (ESCs). The test system uses ESCs at two differentiation stages: undifferentiated ESCs and ESC-derived neurons. Under each condition, concentration–response curves were obtained for three parameters: activity of the *tubulin alpha 1* promoter (typically activated in early neurons), activity of the *elongation factor 1 alpha* promoter (active in all cells), and total DNA content (proportional to the number of surviving cells). We tested 37 compounds from the ESNATS test battery, which includes polypeptide hormones, environmental pollutants (including methylmercury), and clinically used drugs (including valproic acid and tyrosine kinase inhibitors). Different classes of compounds showed distinct concentration–response profiles. Plotting of the lowest observed adverse effect concentrations (LOAEL) of the neuronal promoter activity against the general promoter activity or against cytotoxicity, allowed the differentiation between neurotoxic/DNT substances and non-neurotoxic controls. Reporter activity responses in neurons were more susceptible to neurotoxic compounds than the reporter activities in ESCs from which they were derived. To relate the effective/toxic concentrations found in our study to relevant in vivo concentrations, we used a reverse pharmacokinetic modeling approach for three exemplary compounds (teriflunomide, geldanamycin, abiraterone). The dual luminescence reporter assay described in this study allows high-throughput, and should be particularly useful for the prioritization of the neurotoxic potential of a large number of compounds.

**Electronic supplementary material:**

The online version of this article (doi:10.1007/s00204-016-1690-2) contains supplementary material, which is available to authorized users.

## Introduction

Identification of neurotoxicity or developmental neurotoxicity is an important challenge. Animal tests remain the principal experimental approach. Although the guidelines for testing neurodevelopmental toxicity of compounds (Tsuji and Crofton 2012) rely mainly on in vivo testing, logistic, scientific, and ethical arguments suggest that well-controlled in vitro systems that detect neurodevelopmental toxicity and/or neurotoxicity should be developed (Hartung and Leist 2008; Leist et al. 2008). Stem cell-based technologies are particularly promising in this respect and the EU consortium “embryonic stem cell-based novel alternative tests” (ESNATS www.esnats.eu) was dedicated to the development of such in vitro systems. Several toxicity test systems were developed in the context of this consortium (Leist et al. 2013), including transcriptomics-based assays (Krug et al. 2013b; Zimmer et al. 2011), a neurite extension assay (Krug et al. 2013a), and a neural crest migration assay (Zimmer et al. 2012, 2014). Given the different parameters measured by different neurotoxicity assays, it is likely that the combination of different in vitro tests, i.e., a test battery will be the most promising approach for predictive in vitro testing (Leist et al. 2014; Zimmer et al. 2014).

Generally, two types of neurotoxicity are distinguished: developmental neurotoxicity (DNT), which basically refers to the impact of toxicants on the highly sensitive developing brain, and general neurotoxicity which occurs in the mature brain and peripheral nervous system (Bal-Price et al. 2015; Smirnova et al. 2014). In many cases, compounds, such as methylmercury, that cause developmental neurotoxicity at low exposure levels may also lead to general neurotoxicity at higher exposure levels. However, other compounds, such as valproic acid, that are clearly developmental neurotoxicants show little evidence for toxicity to the mature brain. Noteworthy, identification and classification of compounds in these categories based on currently available studies appear to be uneasy. Major limitations of presently available studies on neurotoxicity, both developmental and general, include the following aspects:

- Relatively few clinical and epidemiological data are available (Smirnova et al. 2014). Well-accepted exceptions are tragic events such as the large-scale methylmercury poisoning in Minamata (Ekino et al. 2007) or the identification of the fetal valproate syndrome because of the widespread treatment of pregnant epileptic patients with valproic acid (Ozkan et al. 2011; Smith and Whitehall 2009);
- Neurotoxicity is typically not characterized by cytotoxicity (i.e., neuronal cell death), but rather by impact of toxicants on connectivity, structure, and function of the nervous system during development or at maturity (Giordano and Costa 2012);
- Extrapolation of data from animal experiments to the human situation may be challenging due to interspecies differences (“humans are no 70 kg mice” (Leist and Hartung 2013);
- In vitro studies on neurotoxicity usually use transformed or cancer cell lines, which have a response pattern significantly different from normal cells (Kadereit et al. 2012; Stiegler et al. 2011).

Validated in vitro methods using stem cell-derived neurons and neural tissues might overcome several of the above cited limitations and allow a more reliable prediction of neurotoxicity (Coecke et al. 2007; Krug et al. 2013b; Pistollato et al. 2012; Rovida et al. 2011; van Thriel et al. 2012). Pluripotent stem cells provide attractive cellular systems for in vitro toxicology studies, because they are non-transformed cells and have the potential to differentiate into the main neural lineages such as neural progenitors cells, neurons, and glial cells (Breier et al. 2010; Kuegler et al. 2010) and provide the starting material for neural tissue engineering (Preynat-Seauve et al. 2009).

The validation of novel in vitro neurotoxicity test systems strongly depends on the availability of well-characterized neurotoxic as well as non-neurotoxic compounds. For this purpose, the ESNATS consortium has developed a compound collection, including the so-called test battery compounds (Zimmer et al. 2014) as well as organomercury compounds and HDAC inhibitors (Krug et al. 2013b). The test battery compound collection includes several classes of compounds, pharmaceuticals (e.g., abiraterone, amiodarone), biologics (e.g., interferon-β, oxytocin), peptide-related small molecules (e.g., sitagliptin, galnon), and environmental pollutants (e.g., PDBE-99, triadimefon). The most widely used model compounds for neurotoxicity are valproic acid and methylmercury. Valproic acid is mostly a developmental neurotoxicant, while methylmercury also causes general neurotoxicity in humans and animals (Kadereit et al. 2012; Wang et al. 2011). When given to pregnant women, the anti-epileptic drug valproic acid causes neural tube defects; in vitro, it triggers relevant changes of the cellular transcriptome through the inhibition of histone deacetylases (HDAC) (Krug et al. 2013b; Theunissen et al. 2011). The mechanisms of action of methylmercury still have not been completely elucidated, but its usefulness as a neurotoxic model compound is undisputed because of the abundantly available clinical data on human neurodevelopmental toxicity and general neurotoxicity (Clarkson and Strain 2003; Davidson et al. 2004; Ekino et al. 2007; Harada 1995). During developmental exposure, methylmercury causes among others neural tube defects (Grandjean and Herz 2011; Robinson et al. 2011), while in the adult exposed to the compound, symptoms such as blurred vision, hearing impairment, olfactory and gustatory disturbances, cerebellar ataxia, somatosensory, and psychiatric disorders were observed (Ekino et al. 2007).

The major limitation of many in vitro neurotoxicity assays is the fact that they are labor-intensive and time-consuming. Mouse embryonic stem cell (mESC) systems show a higher throughput and robustness when compared to the human counterpart, and they offer a better chance to compare in vitro data with the already existing murine and rat in vivo databases (Kuegler et al. 2010; Leist et al. 2013). Furthermore, these systems can be easily engineered for high content imaging (HCI) approaches (van Vliet et al. 2014) or with reporter constructs providing a faster readout. We have previously developed a dual-luciferase reporter construct that upon expression in mouse embryonic stem cells provide read-out in stem cell-derived neurons (Suter et al. 2009). In the context of the ESNATS consortium, we have screened a 1000-compound library with a single fixed concentration per compound, demonstrating the potential of this assay (Kern et al. 2013).

In the present study, we used the dual luminescence reporter assay to establish a high-throughput neurotoxicity test that compares the impact of toxicants on undifferentiated pluripotent stem cells (ESCs) with the impact on differentiated ESC-derived neurons. For each tested compound, the assay determines six dose–response curves: three dose–response curves in undifferentiated ESCs, and three in ESC-derived neurons. To evaluate the human relevance of concentrations tested positive in the dual luminescence reporter assay, a physiology-based pharmacokinetic (PBPK) reverse modeling method was applied. We propose this approach as a promising tool for identifying compounds that may cause DNT/neurotoxicity.

## Materials and methods

### Chemicals

Murine CGR8 ESCs were purchased from European Collection of Cell Culture. The bone marrow stromal MS5 cell line was kindly provided by Katsuhiko Itoh (Itoh et al. 1989). Cell culture reagents were purchased from Gibco, Invitrogen Corporation (Paisley, Scotland). Dual-luciferase^®^ Reporter Assay System was from Promega (Madison, WI, USA), the Fluostar Optima reader from BMG Labtech GmbH (Hanns-Martin-Schleyer-Str. 10, D-7656 Offenburg/Germany), and the Flexstation 3 microplate reader from Molecular Devices (Sunnyvale, California, USA). All non-neurotoxic and cytotoxic controls used in this study were obtained from Sigma. Providers of the other compounds are listed in Table 1.

**Table 1:** The ESNATS compounds collection

Compound	Pharmacological characteristics	Chemical characteristics	Clinical observation	Supplier info	Tested concentrations
	Blockade of voltage-dependent sodium channels; HDAC inhibitor	Carboxylic acid	Krug et al. (2013b), Verrotti et al. (2014)	Sigma-Aldrich	ESC/neurons: 0.005–50 mM
	HDAC inhibitor	Hydroxamic acid	Lawless et al. (2009), Poole (2014)	Selleckchem	ESC/neurons: 0.0005–50 μM
	HDAC inhibitor	Hydroxamic acid	Rasmussen et al. (2015), Richardson et al. (2013)	Selleckchem	ESC/neurons: 0.00005–50 μM
	HDAC inhibitor	Benzamide	Gojo et al. (2007), Kummar et al. (2007)	Enzo Life Science	ESC/neurons: 0.005–50 μM
	Heterogeneous mechanisms (i.e., oxidative stress, disruption of calcium homeostasis, inhibition of protein synthesis)	Organometallic cation	Ekino et al. (2007), Farina et al. (2011), Grandjean and Herz (2015)	Sigma-Aldrich	ESC: 0.005–50 μM neurons: 0.00005–50 μM
	Heterogeneous mechanisms (i.e., disruption of calcium homeostasis, apoptosis, mitochondrial dysfunction, oxidative stress)	Organomercury compound	http://hazmap.nlm.nih.gov/category-details?table=copytblagents&id=2562	Sigma-Aldrich	ESC/neurons: 0.005–10 μM
	ATPase inhibition, Protein inhibition	Organomercury compound	Goshman (1985)	Sigma-Aldrich	ESC/neurons: 0.005–10 μM
	Heterogeneous mechanisms (i.e., mitochondrial toxicity, oxidative stress, inhibition of protein synthesis)	Organomercury compound	Dorea et al. (2013) http://www.who.int/vaccine_safety/committee/topics/thiomersal/statement_jul2006/en/	Sigma-Aldrich	ESC: 0.005–50 μM neurons: 0.0005–50 μM
	Protease inhibitor	Organomercury compound	http://hazmap.nlm.nih.gov/category-details?id=16385&table=copytblagents	Sigma-Aldrich	ESC: 0.005–50 μM neurons: 0.005–100 μM
	Hormone stimulating granulopoiesis	Peptide/Protein	Schneider et al. (2005)	R&D Systems	ESC/neurons: 0.005–2.66 nM
	EpoR agonist controlling erythropoiesis and neurogenesis	Peptide/Protein	Subiros et al. (2012)	R&D Systems	ESC/neurons: 0.5–238 nM
	Activation of Janus kinase and Stat1/2; used for the treatment of relapsing/remitting multiple sclerosis	Peptide/Protein	Plosker (2011)	R&D Systems	ESC/neurons: 0.5–200 pM
	Endogenous agonist of erbB family of tyrosine kinase receptors	Peptide/Protein	Deng et al. (2013)	R&D Systems	ESC/neurons: 0.005–3 nM
	Stimulation of uterine contraction and lactation	Peptide/Protein	MacDonald et al. (2011)	R&D Systems	ESC/neurons: 0.05–100 nM
	Brominated flame retardant acting on calcium homeostasis	Polybrominated diphenyl ether	Costa et al. (2008), Eskenazi et al. (2013), Gascon et al. (2012), Messer (2010)	Sigma-Aldrich	ESC/neurons: 0.005–50 μM
	Environmental toxicant acting on calcium homeostasis	Polychlorinated biphenyl	Grandjean and Landrigan (2006), Jacobson and Jacobson (2001)	Sigma-Aldrich	ESC/neurons: 0.005–25 μM
	Pesticide with teratogenic effects in animals	Triazole	No data available in humans	Sigma-Aldrich	ESC/neurons: 0.005–50 μM
	Inhibition of cyp enzymes and reduction of steroidogenesis	Triazole	http://www.fao.org/fileadmin/templates/agphome/documents/Pests_Pesticides/JMPR/Report10/Cyproconazole.pdf	Sigma-Aldrich	ESC/neurons: 0.005–50 μM
	Active metabolite of the widely used industrial chemical ethylene glycol monomethyl ether	Carboxylic acid	Welsch (2005)	Sigma-Aldrich	ESC/neurons: 0.005–50 μM
	Tyrosine kinase inhibitor	Peptide mimetic	Rinne et al. (2012)	Selleckchem	ESC/neurons: 0.005–10 μM
	Inhibitor of epidermal growth factor receptor tyrosine kinase domain	Nucleotide mimetic	Kim et al. (2008), Maruyama et al. (2008)	Selleckchem	ESC/neurons: 0.005–50 μM
	Tyrosine kinase inhibitor inhibiting signaling of three growth factor receptors involved in angiogenesis	Nucleotide mimetic	Patejdl et al. (2013)	Selleckchem	ESC/neurons: 0.005–10 μM
	Dipeptidyl peptidase-4 inhibitor, oral antidiabetic drug	Peptide mimetic	http://www.ema.europa.eu/docs/en_GB/document_library/EPAR_-_Summary_for_the_public/human/000722/WC500039055.pdf	Selleckchem	ESC/neurons: 0.005–50 μM
	Glucagon-like peptide-1 receptor agonist, antidiabetic drug	Peptide/Protein	Aviles-Olmos et al. (2014)	Prospec	ESC/neurons: 0.005–20 nM
	Thiol groups are the main targets; used to treat a specific type of acute promyelocytic leukemia	Inorganic compound, arsenite (As_2_O_3_)	Grandjean and Herz (2015), Vahidnia et al. (2007)	Sigma-Aldrich	ESC/neurons: 0.005–50 μM
	Affects excitatory and inhibitory synaptic processes; disruption of calcium homeostasis	Organotin compound	Besser et al. (1987), Kreyberg et al. (1992)	Sigma-Aldrich	ESC: 0.005-50 μM neurons: 0.00005–50 μM
	Inhibition of Hsp90 function; antitumor agent	Amide	Kummar et al. (2010)	Selleckchem	ESC/neurons: 0.00005–50 μM
	Antiviral drug for hepatitis C; protease inhibitor	Peptide mimetic	http://www.fda.gov/downloads/AdvisoryCommittees/CommitteesMeetingMaterials/Drugs/AntiviralDrugsAdvisoryCommittee/UCM252561.pdf	Selleckchem	ESC/neurons: 0.005–50 μM
	Antiarrhythmic blocking sodium channels	Tertiary amine	Orr and Ahlskog (2009), Willis and Lugo (2009)	Sigma-Aldrich	ESC/neurons: 0.005–50 μM
	Dopaminergic antagonist with further effects on different systems (adrenergic, serotonergic, cholinergic and histaminergic)	Tertiary amine	Morris et al. (2009)	Sigma-Aldrich	ESC/neurons: 0.005–50 μM
	Immunomodulatory drug which inhibits pyrimidine de novo synthesis	Amide	Lu et al. (2014)	Enzo Life Science	ESC/neurons: 0.005–50 μM
	Antiandrogen inhibiting CYP17A1, an enzyme involved in testosterone synthesis	Steroid	http://www.zytiga.com/sites/default/files/pdf/full_product_information.pdf	Selleckchem	ESC/neurons: 0.005–2 μM
	Selective agonist at galanin receptors; anticonvulsant, anxiolytic in animals	Peptide mimetic	No data available in humans	Bachem	ESC/neurons: 0.005–20 μM
	PDE5 inhibitor; drug used for erectile dysfunction and pulmonary arterial hypertension	Nucleotide mimetic	Campbell et al. (2015)	Sigma-Aldrich	ESC/neurons: 0.005–50 μM
	Antibiotic stopping the production of folic acid in parasites	Benzene sulfonamide	Reboli and Mandler (1992)	Sigma-Aldrich	ESC/neurons: 0.005–50 μM
	Inhibition of renin protease, antihypertensive drug	Peptide mimetic	Daugherty (2008)	Selleckchem	ESC/neurons: 0.005–50 μM
	Inhibition of the factor Xa protease, oral anticoagulant	Peptide mimetic	Abrams and Emerson (2009)	Selleckchem	ESC/neurons: 0.005–10 μM

### CGR8-2Luc cells

**Fig. 1:**
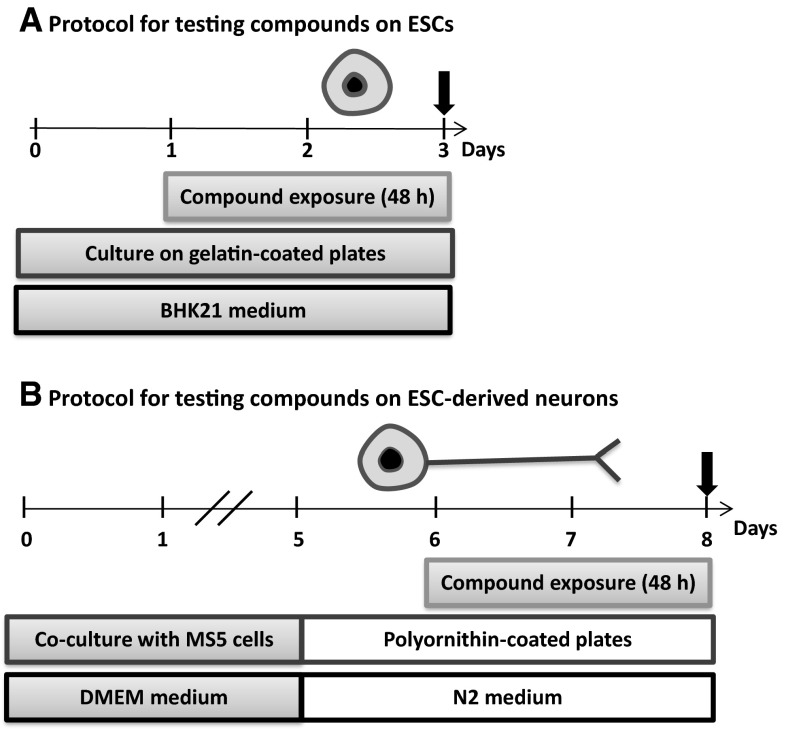
Synopsis of experimental protocols. The impact of compounds on neural promoter activity (FLuc, Firefly Luciferase under the control of the Tα1 promoter), general promoter activity (RLuc, Renilla Luciferase under the control of the EF1α promoter), and amount of DNA (PI, propidium iodide fluorescence in cell homogenates) was investigated in undifferentiated mouse embryonic pluripotent stem cells (ESCs) and on ESC-derived neurons. *Arrows* indicate the time of measurement of the three parameters. **a** *Protocol for testing compounds effects on ESCs.* CGR8-2Luc cells were plated on gelatin-coated 96-well plates in maintenance medium (BHK21 medium containing 10 % FCS, l-glutamine, NEAA, P/S, and LIF). Twenty-four hours later, cells were exposed to compounds for forty-eight hours. **b** *Protocol for testing compounds on ESC*-*derived neurons.* CGR8-2Luc cells were co-cultured with MS5 cells to induce neural differentiation in DMEM medium containing 15 % KO-serum, NEAA, β-mercaptoethanol, and P/S. On day 5, cells were detached and seeded on polyornithine-coated plates in DMEM medium containing N2 supplement, bFGF, and P/S; compound exposure was from day 6 to day 8

Dual luciferase expressing CGR8-2Luc cells were obtained by transduction of mouse ESC CGR8 cells with the 2K7 EFS-Renilla Luciferase (RLuc)/Talpha1-Firefly Luciferase (FLuc) vector as previously described (Suter et al. 2006, 2009); EFS corresponds to the short promoter of the eukaryotic translation elongation factor 1 alpha (EF1α), and Talpha1 to the Tubulin α1 (Tα1) promoter. Tα1 was selected as a promoter active in neurons, including early stages of neural differentiation. EF1α was originally selected to function as a constitutive promoter; however, it responds to cellular differentiation as well as to various chemicals, as previously shown (Kern et al. 2013). Cells were cultured on 0.1 % gelatin-coated dishes in CGR8-2luc maintenance medium: BHK21 medium, supplemented with 10 % fetal calf serum, l-glutamine, non-essential amino acids, penicillin/streptomycin, and leukemia inhibitory factor (LIF). CGR8 cells were grown in a feeder-independent manner (embryonic pluripotent stem cells, ESCs) as described in protocol 1 (Fig. 1) before testing compounds of interest.

### CGR8 2-Luc differentiation and exposure to compounds

To test compounds on ESC-derived neurons, neuronal differentiation was carried out as previously described (Suter et al. 2009; Xu et al. 2014). Briefly, CGR8-2Luc cells were seeded on irradiated bone marrow-derived stromal MS5 cells and cultivated for 4 days in DMEM medium containing 15 % Knock-out™ Serum Replacement, non-essential amino acids, beta-mercaptoethanol, and penicillin/streptomycin. At day 5, cells were then re-plated (3 × 10^5^ cells/cm^2^) on polyornithine-coated 96-well plates in N2 medium containing DMEM, N2 supplement, penicillin/streptomycin, and 10 ng/ml basic human fibroblast growth factor (bFGF) (Invitrogen). After 48 h of incubation in N2 medium, abundant cells with neuron-like morphology were observed. Immunofluorescence analysis (data not shown) demonstrated that 100 % of these cells were βIII tubulin-positive, as previously described (Kern et al. 2013). However, most cells did not yet stain positive for neuronal subtypes (glutamatergic neurons = vGlut (vesicular glutamate transporter), GABAergic neurons = GAD67 (glutamic acid decarboxylase), cholinergic neurons = ChAT (choline acetyltransferase), with the notable exception of some neurons staining for tyrosine hydroxylase (TH) (i.e., a marker of dopaminergic neurons). For toxicity testing, cells were grown for 24 h after replating before exposure to the respective compounds for 48 h. Cells were lysed, and the dual luminescence assay was performed followed by propidium iodide (PI) measurement (protocol 2, Fig. 1). To assess toxicity of compounds on undifferentiated pluripotent stem cells (ESCs), CGR8-2Luc cells were plated at 45,000 cells/cm^2^ on 0.1 % gelatin-coated dishes in BHK21 medium, supplemented with 10 % fetal calf serum, l-glutamine, non-essential amino acids, penicillin/streptomycin, and leukemia inhibitory factor (LIF). After 24 h, compounds were added and incubated for 48 h, before luciferase expression and PI fluorescence were analyzed. In all experiments, methylmercury was used as a positive control at a concentration of 5 µM.

### Dual luminescence assay

Luciferase activities were measured with a Dual-Luciferase™ Reporter system kit. CGR8-2Luc ES cells were lysed in 96-well plates according to the manufacturer’s instructions. Luminescence measurements were performed on a Fluostar Optima reader. Luminescence counts were expressed as percentage of promoter activity normalized by comparison with the control wells treated by DMSO. High standard deviation (SD) observed in RLuc values is explained by the very low expression of the general promoter in neurons. Two parameters were measured in homogenates of ESCs and in ESC-derived neurons: (1) FLuc, reflecting the activity of early neural promoter Tα1. FLuc increases during neuronal differentiation. Many neurotoxic compounds may affect FLuc expression, either through impact on neuronal differentiation, through changes of neuronal gene expression, or through killing of neurons. Note, however, that in some cases, there may be an increase of FLuc expression through potential neurotoxicants (Kern et al. 2013); (2) RLuc, reflecting the activity of the ubiquitous promoter EF1α. Note that in previous studies we have shown that this promoter activity markedly decreased during cellular differentiation and it can therefore not be used as a house-keeping gene to approximate the cell numbers during cellular differentiation.

### Propidium iodide (PI) assay

After measurement of luciferase activity, DNA quantity was determined by PI assay, as previously described (Xu et al. 2014). PI was added to cell homogenates after the luciferase test, at a final concentration of 50 µg/ml and incubated for 2 h. After incubation, fluorescence intensity was measured on a Flexstation 3 microplate reader (Excitation: 544 nm ± 15 nm; Emission: 620 nm ± 15 nm). Results are expressed as percentage of control. Propidium iodide (PI) measures total DNA content as an approximation of the cell number. A decrease in PI fluorescence was interpreted as a decrease in cell number and therefore throughout the text referred to as cytotoxicity. Note that none of the compounds led to an increase in PI fluorescence.

### Statistical analysis

Luminescence counts were expressed as percentage of promoter expression normalized by comparison with the control wells treated by DMSO. PI results are expressed as percentage of control. For outlier analysis, we performed the Grubbs’ test. The results were analyzed using GraphPad Prism 6 software (GraphPad Software, San Diego, CA).

### Assay performance and plate acceptance criteria

To monitor assay sensitivity, S/B ratios were calculated as mean of negative control signal/mean of positive control. Mean, SD, and coefficient of variation (CV) for each signal (positive and negative controls) were computed. As acceptance criteria, we set a maximum CV of each signal at 20 %. To evaluate the robustness of each assay, we assessed the *Z* value and the Signal Window (SW) calculated according to the following equations: *Z* = 1−(3SD of negative control + 3SD of positive control)/|(mean of positive control–mean of negative control)| and SW = ((mean negative control–3SD negative control/√*n*)–(mean positive control + 3SD positive control/√*n*))/(SD negative control/√*n*) (Iversen et al. 2012; Perrin et al. 2006), where *n* is the number of replicates of the test substance that has been used in our assay. As acceptance criteria, we chose SW ≥ 3 and *Z* ≥ 0.4 on all plates.

### Characterization of concentration–response curves

To describe the shape of different concentration–response curves, arbitrary scores were assigned as follows: 0 for “no change,” 1 for “down-stroke,” 2 for “bimodal profile,” and 3 for “up-stroke” (Fig. S1). A curve was classified as “down-stroke” or “up-stroke” if the change in response values comparative to control was statistically significant (*p* value <0.05, one-way ANOVA with post hoc Dunnett) with an additional requirement of a 30 % decrease or increase of mean response value relative to controls, respectively. If the absolute change in mean response values was below 30 % the curve was classified as “no change.” A curve was classified as “bimodal” if first up-stroke and subsequently down-stroke was observed.

### LOAEL evaluation

Lowest adverse effect level (LOAEL) was defined as the lowest tested concentrations that lead to a statistically significant decrease for a given read-out (FLuc, RLuc, and PI) compared to baseline. “Baseline” was not in all cases defined by untreated cells, but may also refer to, e.g., a state of increased activity due to low, non-toxic compound concentrations.

### In vitro–in vivo comparison of toxicity data by PBPK modeling

To evaluate the human relevance of in vitro concentrations found to be toxic in this study, an in vitro–in vivo comparison of the toxicity data was performed using a physiology-based pharmacokinetic (PBPK) reverse modeling approach. In particular, the following steps were taken: (a) data mining to find published studies reporting relevant concentrations inducing (neuro-) developmental toxicity in vivo or reporting relevant therapeutic concentrations reached in humans during clinical studies (when possible); (b) extraction of pharmacokinetic (PK) parameters from published studies in rats or humans and use of these data to calculate free plasma concentrations; (c) calculation of the nominal in vitro concentrations equivalent to the concentrations predicted in vivo (NEC), determined by correcting for the differences in albumin concentration and lipid fraction between plasma and test medium, using the following equation:

$${\text{NEC}} = EC_{pl} \times \left\{ {\left( {1 - f_{b,pl} } \right) \times \frac{{1 + Kow \times VF_{L,x} }}{{1 + Kow \times VF_{L,pl} }} + f_{b,pl} \times \frac{{P_{x} }}{{P_{pl} }}} \right\}$$

where *EC*_*pl*_ is the effective plasma concentration; *f*_*b,pl*_ corresponds to the plasma bound fraction; *K*_*ow*_ is the octanol:water partition coefficient; *VF*_*L*_ is the volume fractions of lipids; *P* corresponds to the albumin concentration (or total protein concentration when indicated); and the suffix “*pl*” and “*x*” are indicating plasma and one of the media used in this study (Gulden and Seibert 2003) (Fig. 7). The lipid content and albumin concentrations of the test media N2 and BHK medium were calculated on the basis of the available information provided by the suppliers (Fig. S2A). The data on rat plasma have been adopted from (Verwei et al. 2006). The original references are (Barber et al. 1990) for albumin and (DeJongh et al. 1997) for lipids. Human plasma values were taken from (Gulden and Seibert 2003). The original data on albumin are from (Lindup 1987) and for lipids from (Patterson et al. 1988). Total protein concentrations were calculated based on the assumption that the protein molar mass is similar to the one of albumin (66 kDa). Albumin was assumed to represent 60 and 48 % of the total serum proteins (mg/ml) in human and rat (Baker et al. 1979; Busher 1990) (Fig. S2A).

### In vitro–in vivo comparison of toxicity/clinical data for abiraterone

(a) Developmental toxicity (DT)—inducing concentrations were extrapolated from the in vivo study reported in the document by the Australian Therapeutic Goods Administration (Australian Therapeutic Goods Administration 2014). In an embryo-fetal developmental toxicity study in rats, abiraterone acetate induced developmental toxicity when administered at oral doses of 10, 30 or 100 mg/kg/day throughout the period of organogenesis (gestational days 6–17). Findings include embryo-fetal lethality (increased postimplantation loss and decreased number of living fetuses), fetal developmental delay (skeletal effects), and urogenital effects (bilateral ureter dilation) at doses ≥10 mg/kg/day (Australian Therapeutic Goods Administration 2014). (b) Toxicokinetic parameters of abiraterone in rats were extracted from the available report from the TGA (Australian Therapeutic Goods Administration 2014). The maximal concentration (Cmax) and AUC at the toxic dose level (10 mg/kg/day) in rats was 10.8 ng/ml and 34 h*ng/ml, respectively. The toxicokinetic parameters of abiraterone in humans were extracted from the study by (Goldberg and Berrios-Colon 2013); in this clinical research, a daily oral dose of 1000 mg/kg, administered as abiraterone acetate and bioactivated by hydrolysis to abiraterone, led to a Cmax of 226 ng/ml and an AUC at steady state of 1173 h*ng/ml. The average concentration (Cavg) was calculated from the ratio of AUC and the dose interval (τ) of 24 h. The percentage of plasma protein binding of the drug was reported as being 99.8 %, without relevant species differences occurring (US FDA 2011). (c) The nominal in vitro concentrations equivalent to the concentration used in vivo were calculated using the value of total protein concentration instead of albumin concentration. This approach was chosen because abiraterone shows extreme lipophilicity and extensive protein binding in vivo; this evidences lead to the assumption that in the absence of albumin and α1-acid glycoprotein in the test medium, abiraterone would bind to other present proteins (Fig. S2B).

### In vitro–in vivo comparison of data for geldanamycin (GA)

(a) No developmental toxicity studies were found for GA in vivo, but different studies reported effects of this drug on neurodifferentiation. We used the study of (Sun et al. 2012) to define a dose that is acting on neurons in vivo. A GA dose of 0.2 mg/kg/day affected nerve recovery in a model based on Thy1-GFP transgenic rats, in which the green fluorescent protein GFP was expressed under the neuron-specific promoter Thy1, allowing to determine the rate of axon regeneration after a nerve injury.

(b) Very few PK data are available for GA. The aqueous solubility of GA is poor, limiting the routes of administration to intravenous and intracerebroventricular. The i.v. PK of GA was studied in mice and dogs (Supko et al. 1995). At the maximum tolerated dose, plasma levels rapidly declined to below-effective concentrations in both species. Interspecies differences in PK in this study limit its usability to estimate rat PK. Based on the initial volume of distribution in mouse of 0.16 l/kg, the dose of 0.2 mg/kg (rat study) would lead to a Cmax of 1.25 mg/l. Supko el al (Supko et al. 1995) noted that the low distribution volume despite its lipophilicity suggests that the compound has a much greater affinity for plasma protein than for interaction with peripheral tissue. We assumed a *f*_*b,pl*_ of 99 % since no more specific indications of plasma protein binding were found. (c) The nominal in vitro concentrations equivalent to the Cmax of 1.25 mg/l in rats were calculated assuming GA binding only to the specific drug-binding proteins (albumin and/or α1-acid glycoprotein) (Fig. S2C).

### In vitro–in vivo comparison of toxicity data for teriflunomide (TF)

(a) TF has been showed to lead to teratogenicity when administered to Sprague Dawley rats at an oral dose of 0.3 mg/kg/day from gestational day (GD) 6 through lactational day (LD) 20. Clinical signs included malrotated forepaws/hindpaws, discoloration of the body surface, impaired coat growth, eye opacity, and eye discharge with absence of pupillary reflex were observed (US FDA 2012). (b) Assuming the toxicokinetic parameters are proportional to reported kinetics at 1 mg/kg (US FDA 2012), Cmax and AUC at 0.3 mg/kg were estimated to be approximately 3 μg/ml and 33 h*μg/ml, respectively. From the clinical point of view, a daily oral dose of 14 mg led to a 24 h AUC at steady state of 1070 h*µg/ml in healthy individuals treated with TF (Australian Therapeutic Goods Administration 2013). The Cavg was calculated as the ratio of AUC and the dose interval (τ) of 24 h. TF is reported to be extensively bound in plasma, probably mostly to albumin, with an average bound fraction of 99.46 % (Russo et al. 2013). (c) The nominal in vitro concentrations equivalent to the concentration used in vivo in rats and humans (Cmax and Cavg) were determined assuming only binding to the specific drug-binding proteins (albumin and/or α1-acid glycoprotein) (Fig. S2D).

## Results and discussion

### Assay establishment

**Fig. 2:**
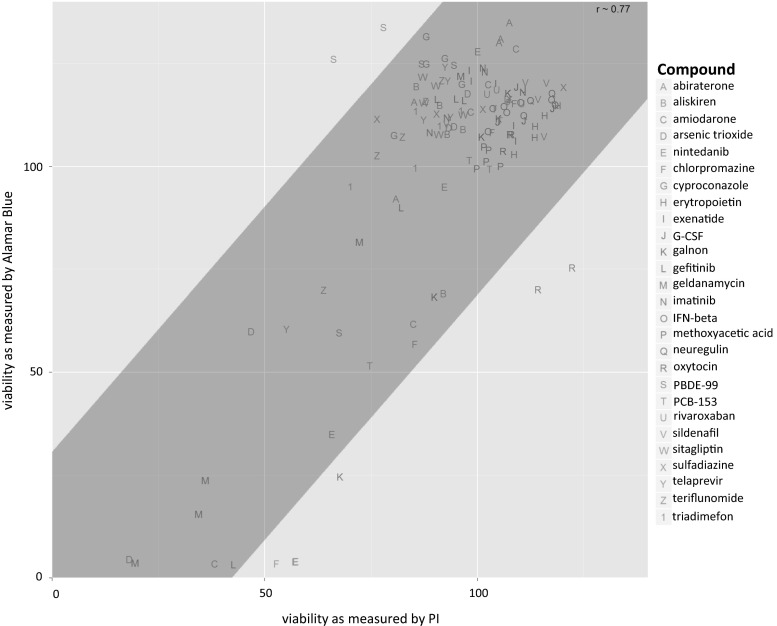
Comparison of two viability assays. To validate the PI assay in the context of toxicity assessment, the cells were exposed to compounds with varying concentrations where the viability of the cells was measured with PI and Alamar Blue assays. The correlation between the two viability assays, that included all compounds and all concentrations, was calculated using Pearson’s correlation. The correlations for each individual data point were plotted, i.e., not the IC50s, but several concentrations for each compound. In addition a linear regression model was fit to the data where the *gray box* indicates the 2 standard deviation of the residuals from the *regression line*

**Fig. 3:**
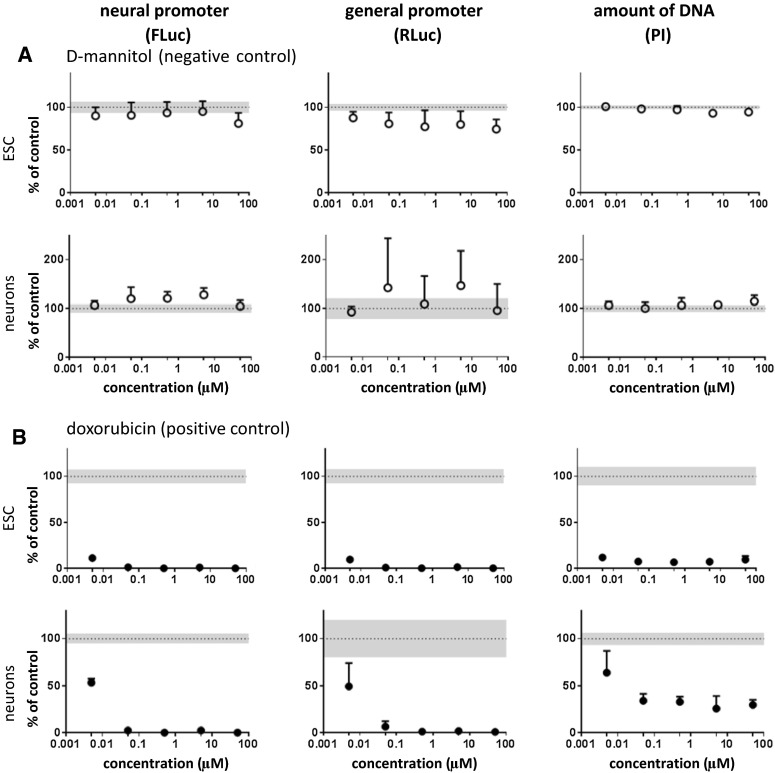
Effect of well-known non-neurotoxic and cytotoxic controls. Cells were exposed to compounds for 48 h; neural (Tα1; FLuc) and general (EF1α; RLuc) promoter activities and DNA quantity (PI assay) were determined. Results were expressed as percent of control + SD. Mean control values (100 %) are shown as *dotted line*; the SD of control values is shown as *gray area*. Data points that differ in a statistically significant manner from control values were determined by one-way repeated-measures ANOVA followed by Dunnett’s post hoc test and are shown as *filled circles*. Data were obtained from 4 to 6 replicates

In this study, a high-throughput dual luminescence reporter assay was established. It compares the impact of test compounds on undifferentiated pluripotent stem cells (ESCs) and ESC-derived neurons (Fig. 1). In both cell types activity of a neuron-specific promoter, *tubulin α1* (T*α1)* and a general promoter, *elongation factor 1α* (EF*1α)* were determined. Moreover, total DNA content was analyzed by the PI assay as a measure of cell number. In contrast to the Alamar Blue method, a technique frequently applied in cytotoxicity testing, the PI assay can be easily integrated into the experimental procedure of the dual luminescence reporter test system. For validation of this new surrogate cytotoxicity endpoint (PI), we compared cytotoxicity data obtained by both assays, Alamar Blue and PI, using all test compounds later analyzed in this study. A high degree of correlation was found between cytotoxicity obtained by both assays (Fig. 2). Therefore, the PI assay was used in all further experiments. In a preliminary study, D-mannitol was tested as a negative control compound and compared to the cytotoxic drug doxorubicin. D-mannitol remained negative up to the highest tested concentration of 50 µM for all three parameters analyzed in undifferentiated ESCs and neurons (Fig. 3a). Other non-neurotoxic controls, saccharin, ibuprofen, omeprazole, nicotinic acid, uric acid, and propranolol were studied and also yielded negative results (Fig. S3, Fig. S4). In contrast, doxorubicin strongly decreased the neuronal and general reporter activities, as well as the cell number (Fig. 3b).

### Response profiles in vitro and the relationship to in vivo relevant concentrations

We next tested a set of 37 compounds to obtain an overview over concentration–response principles of the neuronal as well as the general promoter: 28 of the tested compounds belonged to the ESNATS test compound battery, including clinically used drugs (e.g., teriflunomide, abiraterone), environmental pollutants (e.g., PCB, PBDE, arsenic), and biologics (e.g., interferon-β, oxytocin). Ten further compounds were HDAC inhibitors (e.g., valproic acid, entinostat) and organomercury compounds (e.g., methylmercury, thimerosal). For some of these compounds, human data were available (Table 1) and could be used to group the compounds into three classes: strong, weak or absent neurotoxicity (Table S1).

**Fig. 4:**
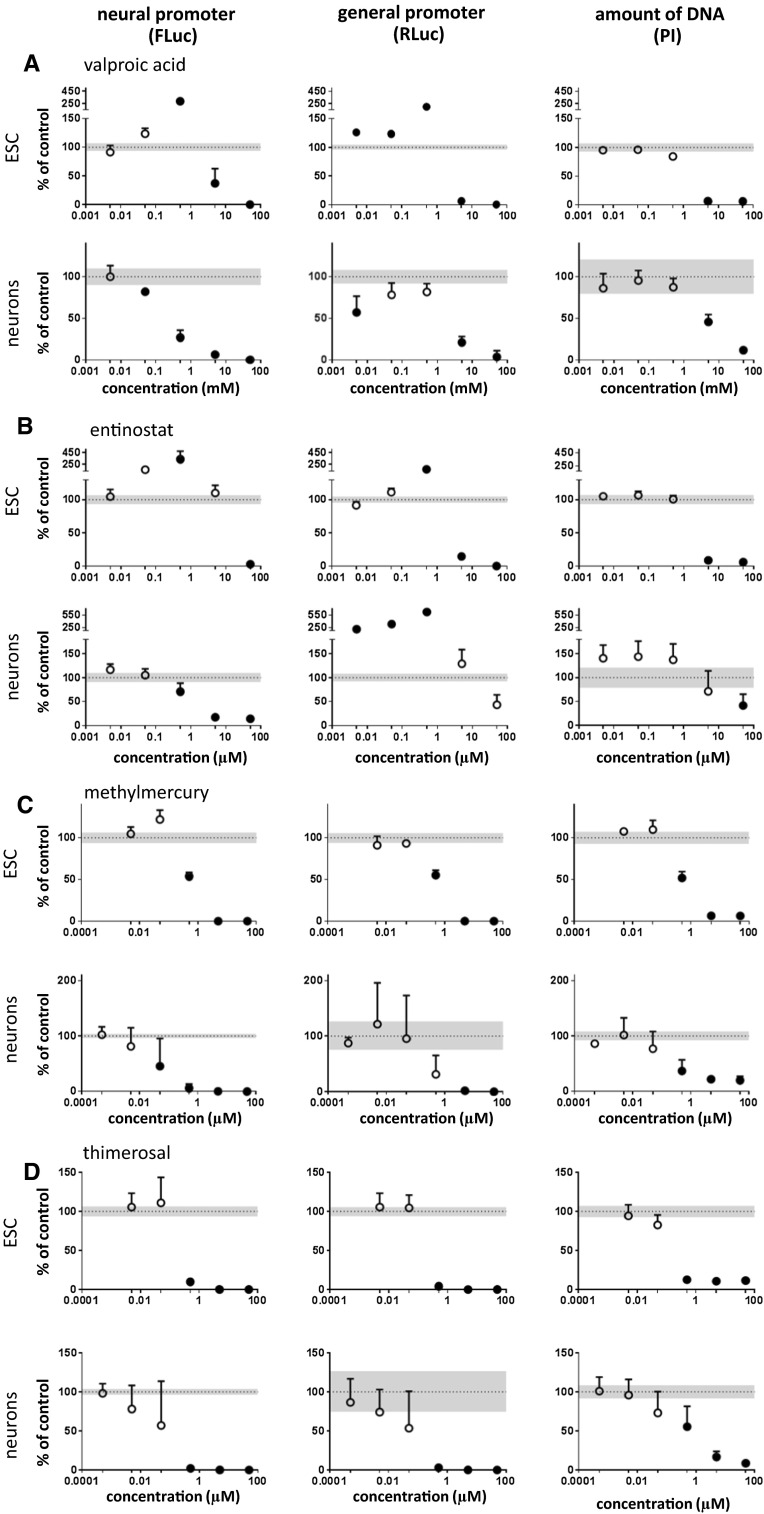
Effect of HDAC inhibitors and organomercury compounds. Cells were exposed to compounds for 48 h; neural (Tα1; FLuc) and general (EF1α; RLuc) promoter activities and DNA quantity (PI assay) were determined. Results were expressed as percent of control + SD. Mean control values (100 %) are shown as *dotted line*; the SD of control values is shown as *gray area*. Data points that differ in a statistically significant manner from control values were determined by one-way repeated-measures ANOVA followed by Dunnett’s post hoc test and are shown as *filled circles*. Data were obtained from 4 to 8 replicates

In the dual luminescence reporter test system, all HDAC inhibitors showed cytotoxicity at their highest test concentrations as evidenced by the PI assay (Fig. 4, Fig. S5A, B). Cytotoxic effects in ESCs occurred at lower concentrations compared to neurons. At non-cytotoxic concentrations, HDAC inhibitors showed distinct effects. In undifferentiated cells, valproic acid, belinostat, and entinostat enhanced the activities of neuronal and/or general promoters (Fig. 4a, b, Fig. S5B). In contrast, in neurons, all HDAC inhibitors showed inhibition of the neural promoter at non-cytotoxic concentrations. Two of the HDAC inhibitors had slightly deviating properties. Entinostat also enhanced general promoter activity in neurons (Fig. 4b), and panobinostat showed no enhancement of promoter activities under any condition (Fig. S5A). Among the inhibitors tested, panobinostat had the most marked inhibitory effects, in particular in neurons where the neuronal promoter was inhibited at subnanomolar concentrations. Note that methoxyacetic acid is the major human metabolite of the environmental toxicant ethylene glycol monomethyl ether (EGME). It has been reported to also show some HDAC inhibitor activity (in the millimolar range). In accordance with this, it yielded only a very weak signal at the highest concentration (micromolar range) tested here (Fig. S6A). Taken together, a typical feature of HDAC inhibitors in our experimental system is their capacity to enhance promoter activities, typically at relatively low concentrations, which are presumably of relevance for in vivo neurotoxicity. The increase in both reporter gene activities could not be ascribed to an increase in cell number, as demonstrated by PI assay, but is the result of an increased promoter activity.

Among the four HDAC inhibitors tested in this study, only valproic acid was a well-characterized human developmental neurotoxicant. The other HDAC inhibitors, which are structurally unrelated to valproic acid are drugs under development for cancer indications (Cheng et al. 2015; Foss et al. 2015; Ruiz et al. 2015). In our dual luminescence reporter test system, the HDAC inhibitors shared similarities but also showed distinct features. Consistent with the role of histone acetylation in epigenetic regulation (Stefanska and MacEwan 2015; Varela et al. 2013), all HDAC inhibitors led to some degree of enhanced reporter activity. However, distinct features were the preferential effects in either ESCs as compared to neurons. Moreover, some HDAC inhibitors preferentially activated either the Tα1 or the EF1α promoter. One common feature of several HDAC inhibitors is the inhibition of the Tα1 promoter in neurons at very low concentrations. Indeed, for valproic acid, statistically significant inhibition of Tα1-driven FLuc expression in neurons was already observed at a concentration of 50 µM. This concentration is of in vivo relevance, since human blood concentrations of valproic acid range between 500 and 1000 µM (Krug et al. 2013b). Previous studies have shown that valproic acid inhibits neural crest cell migration in the 10–100 μM range without affecting neuroepithelial precursor cell migration even at concentrations of 1 mM (Zimmer et al. 2012). The results of valproic acid of the present study are in good agreement with previous studies performed in neuronal precursor cells derived from human embryonic stem cells (Waldmann et al. 2014). In this study, three types of impacts related to different concentration ranges have been defined: (1) a range of tolerance, observed valproic acid concentrations up to 25 μM, suggesting the existence a threshold mechanisms (Dietrich et al. 2013); (2) a deregulated/teratogenic effect was observed at concentrations between 150 and 550 μM of valproic acid, which was associated with developmental disturbances, impaired cell migration, and the down-regulation of neuronal pathways (Balmer et al. 2012; Klaric et al. 2013); (3) a cytotoxic concentration range at 800 and 1000 μM.

While valproic acid is known as a DNT compound, methylmercury represents a model compound that triggers both developmental and adult neurotoxicity in humans and animals (Grandjean and Landrigan 2006; Kadereit et al. 2012). In the present study, four organomercury compounds have been tested (phenyl-mercuric acetate, thimerosal, 4-chloromercuric benzoic acid, and mercury bromide) in addition to methylmercury chloride. All organomercury compounds showed a very similar profile: a marked decrease in the three measured parameters, neural and general promoter activities, and total DNA content, on both ESCs and ESC-derived neurons (Fig. 4c, d, Fig. S5C, D and Fig. S7A). However, mercury bromide had less effect on neurons, as compared to ESCs. Up to concentration of 5 µM, neurons were not affected by mercury bromide, while the compound appeared already highly cytotoxic on ESCs at this concentration (Fig. S5C). Strong differences in the toxicities of closely related mercurials are well documented in the literature (Lohren et al. 2015; Rempel et al. 2015).

At first glance, it may seem surprising that the well-known neurotoxicant methylmercury did not show a strong preferential neurotoxicity, as judged by comparison of its effects on the activity of the neural promoter vs the general promoter; or on comparing the toxicity to neurons vs ESCs. However, as noticed previously by others (Silva-Pereira et al. 2005; Suñol and Rodríguez-Farré 2012; van Vliet et al. 2008), the in vitro toxicity of methylmercury typically includes a strong cytotoxic component. Yet, methylmercury showed a statistically significant inhibition of Tα1 promoter activity in neurons already at 0.05 µM, while significant changes in the other parameters and in ESCs were observed only at higher concentrations in the range between 0.5 and 5 µM. It should also be noted that human relevant concentrations for methylmercury are in the range of 0.005–0.5 µM (Krug et al. 2013b). Thus, the influence of methylmercury on Tα1 promoter activity in neurons occurs at in vivo relevant concentrations and seems to represent an adequate marker of neurotoxicity.

Although the use of organomercury compounds such as fungicides was banned in the early 1970s (Westermark et al. 1975) and its use as an antimicrobial agent was significantly decreased or banned in many countries, mercury is currently employed as a preservative, thimerosal, in multi-dose vials of some vaccines, which are prescribed to pregnant mothers and infants (Dorea et al. 2013). It is metabolized to the cell-permeant ethylmercury in the human body and may therefore have neurotoxic effects through binding to intracellular targets. In our assay system, statistically significant effects of thimerosal on the reporter genes in neurons were observed only at concentrations of 0.5 µM (Fig. 4d). In vivo, a small increase in blood mercury levels (<5 ng/ml, which is approximatively 25 nM) after vaccination has been reported (Pichichero et al. 2008), which is well below the concentrations leading to statistically significant effects in our assay system. This fits well with the available epidemiological studies, which have rejected a causal relationship between thimerosal-containing vaccines and autism or neuropsychological functioning (Hurley et al. 2010; Thompson et al. 2007). Indeed, a statement was issued by the WHO in 2006 that there is no scientific evidence in favor of a neurotoxic/DNT effect of thimerosal in babies, children, or adults exposed to the compound by way of vaccination (http://www.who.int/vaccine_safety/committee/topics/thiomersal/statement_jul2006/en/).

**Fig. 5:**
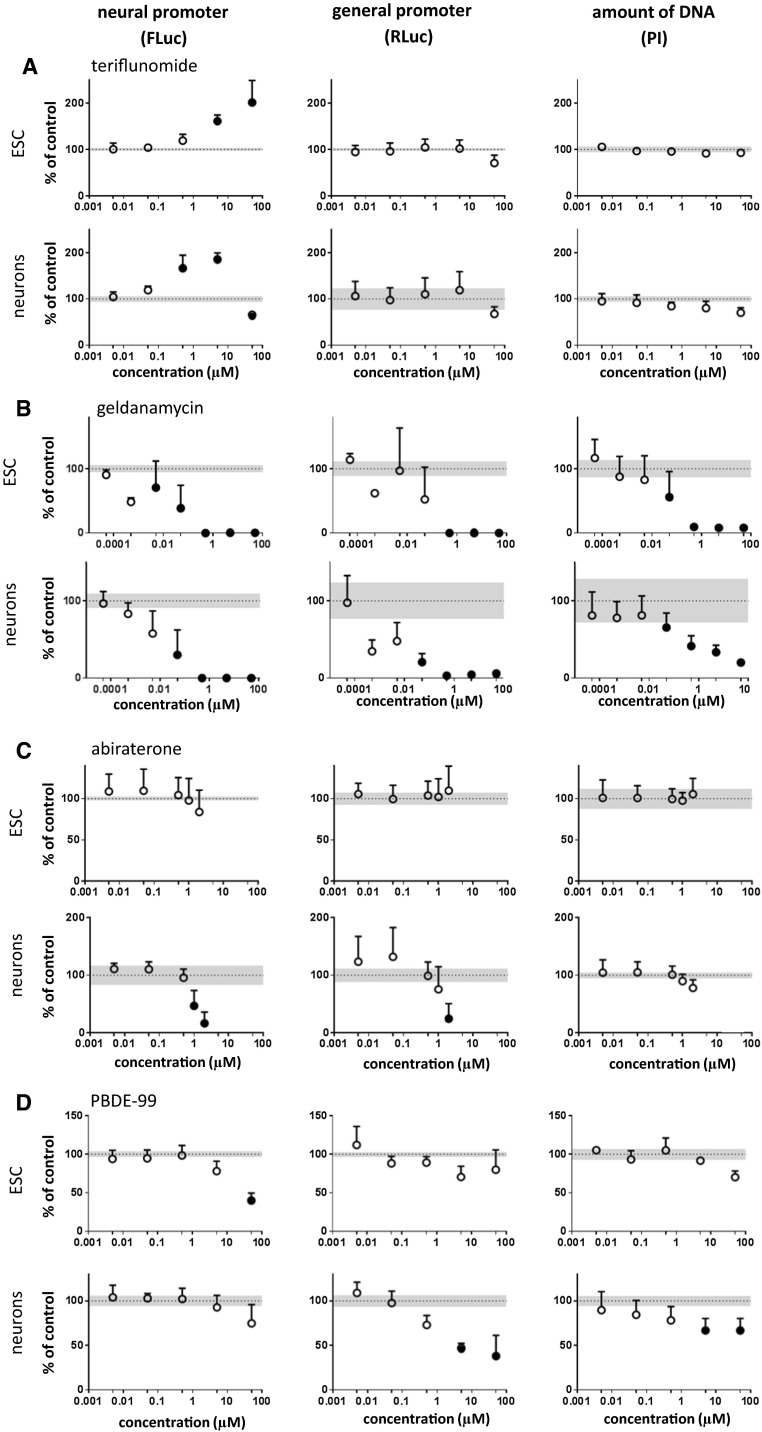
Representative examples of different classes of tested compounds. Cells were exposed to compounds for 48 h; neural (Tα1; FLuc) and general (EF1α; RLuc) promoter activities and DNA quantity (PI assay) were determined. Results were expressed as percent of control + SD. Mean control values (100 %) are shown as *dotted line*; the SD of control values is shown as *gray area*. Data points that differ in a statistically significant manner from control values were determined by one-way repeated-measures ANOVA followed by Dunnett’s post hoc test and are shown as *filled circles*. Data were obtained from 4 to 12 replicates

The immunomodulatory drug teriflunomide is known for its teratogenicity without documented neurotoxicity. In our experiments, it had no effects on general promoter activity and/or on PI fluorescence. However, it caused an increase in neuronal promoter activity both in neurons and in ESCs. The increase of FLuc activity may reflect its ability to enhance neural differentiation. Alternatively, it may suggest that the compound interferes with epigenetic regulation, similarly as seen above for the HDAC inhibitors. While this enhancement of promoter activity was monophasic in ESCs, it was a biphasic curve with an upstroke between 0.05 and 0.5 µM, followed by a downward deflection at a concentration of 50 µM in ESC-derived neurons (Fig. 5a). Thus, our results suggest that at very high concentrations (50 µM), teriflunomide may lead to neurotoxicity, while neurodevelopment may be affected at lower concentrations already.

Geldanamycin is a benzoquinone ansamycin antibiotic that inhibits the function of Hsp90 (Fukuyo et al. 2010). It is used as an experimental anticancer agent in animal experiments. Presently, there is no evidence for neurotoxicity of the compound. However, several previous studies have reported in vitro cytotoxicity (Clark et al. 2009; Mlejnek and Dolezel 2014; Wu et al. 2010). In our study, the compound showed a considerable cytotoxicity on neurons and on ESCs. This cytotoxicity was already observed at concentrations of 0.05 µM (Fig. 5b). Thus, the main effect of geldanamycin in our experimental system is cytotoxicity, and the impact on the reporter genes should be rather considered as secondary to this cytotoxicity. Interestingly, the LD50 of geldanamycin in mice is 1 mg/kg (https://www.spectrumchemical.com/MSDS/TCI-G0334.pdf), thus relatively low and in line with the observed cytotoxicity.

Abiraterone is an anti-androgenic drug for therapy of some metastatic cancers (Patel 2013) without known neurotoxicity. Preclinical studies reported developmental toxicity in rats (Australian Therapeutic Goods Administration 2014). In our assay system, abiraterone showed no cytotoxicity up to concentrations of 100 µM in the PI assay. In absence of changes in cell number, it induced a decrease of neuronal and general promoter activities in ESC-derived neurons (Fig. 5c) suggesting that at concentrations higher than 0.5 µM, this drug affects neural differentiation. The relationship of this concentration to clinically relevant ranges will be discussed below.

Amiodarone is a class III anti-arrhythmic drug generally prescribed for atrial fibrillation and ventricular arrhythmias. The early published experience with amiodarone suggested that neurotoxic effects such as ataxia, peripheral neuropathy, and cognitive impairment/encephalopathy were frequent (Charness et al. 1984; Greene et al. 1983). However, clinically significant neurotoxic effects seem to be observed only at high concentrations, well above those achieved with presently used drug doses (Orr and Ahlskog 2009). In a previous study searching for neurotoxic and neuroactive compounds, we observed that amiodarone clustered with other potentially neurotoxic drugs; however, only a high concentration (10 µM) was tested (Kern et al. 2013). In this study, we found that the LOAEL of amiodarone in our experimental system was 5 µM (FLuc in neurons; Fig. S7C, Table 2). Concentrations of amiodarone in humans have been estimated in the range of 2–3 µM (Table 2). Thus, amiodarone provides an interesting example of a potentially neurotoxic compound where achievable drug levels approach the neurotoxicity threshold.

**Table 2:** Lowest adverse effect levels (LOAEL) and the concentrations expected to be found in humans (when available) across all compounds

Class	Compounds	LOAEL (µM)						Concentration in humans (µM)	Number of non-cytotoxic changes
		ESC Tα1	ESC EF1α	ESC DNA	Neurons Tα1	Neurons EF1α	Neurons DNA		
Non-neurotoxic controls	D-mannitol	>50	>50	>50	>50	>50	>50		0
	Ibuprofen	>50	>50	>50	>50	>50	>50	150^a^	0
	Omeprazole	>50	>50	>50	>50	>50	>50	0.8^b^	0
	Nicotinic acid	>50	>50	>50	>50	>50	>50		0
	Uric acid	>50	>50	>50	>50	>50	>50		0
	Saccharin	>50	>50	>50	>50	>50	>50	218^c^	0
	Propranolol	50	50	>50	50	>50	50	0.12–0.19^d^	2
Cytotoxic control	Doxorubicin	0.005	0.005	0.005	0.005	0.005	0.005		0
HDAC inhibitors	Valproic acid	5000	5000	5000	50	5000	5000	500–1000^e^	2
	Belinostat	5	5	0.5	0.0005	50	50	0.36^f^	1
	Entinostat	50	5	5	0.5	>50	50	0.3^g^	1
	Panobinostat	0.05	0.05	0.05	0.0005	50	0.05	0.036–0.045^h^	1
Organomercury compounds	Methylmercury	0.5	0.5	0.5	0.05	5	0.5	0.005–0.5^e^	1
	Phenylmercuric acetate	0.5	0.5	0.5	0.5	0.5	0.5	5–50 mg/kg (oral lethal dose)^i^	0
	Thimerosal	0.5	0.5	0.5	0.5	0.5	0.5	Max 5 ng/ml^j^	0
	4-chloromercuric benzoic acid	5	5	5	10	100	100		1
	Mercury bromide	5	5	5	10	>10	>10		1
Polypeptides	G-CSF	>2.66*10^−3^	>2.66*10^−3^	>2.66*10^−3^	>2.66*10^−3^	>2.66*10^−3^	>2.66*10^−3^	1.3*10^−6k^	0
	Erythropoietin	>0.238	>0.238	>0.238	>0.238	>0.238	>0.238	0.0003^k^	0
	IFN-β	>2*10^−4^	>2*10^−4^	>2*10^−4^	>2*10^−4^	>2*10^−4^	>2*10^−4^	0.4*10^−6^–7.5*10^−3k^	0
	Neuregulin	>3*10^−3^	>3*10^−3^	>3*10^−3^	>3*10^−3^	>3*10^−3^	>3*10^−3^	6.3^k^	0
	Oxytocin	>0.1	>0.1	>0.1	>0.1	>0.1	>0.1	0.002^k^	0
Environmental pollutants	PBDE-99	50	>50	>50	>50	5	5	97*10^−6k ^(human exposure concentration)	1
	PCB-153	>50	>50	>50	50	50	>50	0.0003-0.002^k^ (human exposure concentration)	2
	Triadimefon	50	>50	>50	>50	50	>50		2
	Cyproconazole	>50	>50	>50	>50	>50	>50		0
	Methoxyacetic acid	>50	>50	>50	50	>50	>50	60^k^ (human exposure concentration)	1
Tyrosine kinase inhibitors	Imatinib	10	>10	>10	10	10	>10	3.0^k^	3
	Gefitinib	50	50	50	50	50	50	1.2^k^	0
	Nintedanib	0.5	5	5	0.05	5	5	0.074^k^	2
Clinically used drugs	Sitagliptin	>50	>50	>50	>50	>50	>50	1.9^k^	0
	Exenatide	>0.02	>0.02	>0.02	>0.02	>0.02	>0.02	0.045^k^	0
	Telaprevir	50	50	50	50	50	50	5.1^k^	0
	Amiodarone	500	500	500	50	500	500	2.3^k^	1
	Chlorpromazine	20	20	20	1	2	20	0.39^k^	1
	Teriflunomide	>50	>50	>50	50	>50	>50	10.8^k^	1
	Abiraterone	>2	>2	>2	1	2	>2	0.65^k^	1
	Sulfadiazine	>50	>50	>50	>50	>50	>50	320^k^	0
	Sildenafil	>50	>50	>50	>50	>50	>50	0.221^k^	0
	Aliskiren	>50	>50	>50	>50	>50	>50	0.33^k^	0
	Rivaroxaban	>10	>10	>10	>10	>10	>10	0.69^k^	0
Broad toxics	Arsenic trioxide	0.5	0.5	0.5	5	5	5	0.2–1.1^k^ (human exposure concentration)	0
	Trimethyltin chloride	5	5	5	5	0.05	5		1
Other compounds	Galnon	20	20	20	5	5	>20		2
	Geldanamycin	0.005	0.5	0.05	0.05	0.05	0.05	0.8^k^	1

Column 10 gives the number of reporter activity changes observed in the non-cytotoxic concentration range for a given compound

Neurons Tα1 (Neurons neural promoter Tub1α); Neurons EF1α (Neurons general promoter EF1α); Neurons DNA (neurons total DNA content); ESC Tα1 (ESC neural promoter Tub1α); ESC EF1α (ESC general promoter EF1α); ESC DNA (ESC total DNA content)

^a^Dewland et al. (2009)

^b^Linden et al. (2007)

^c^Sweatman et al. (1981)

^d^International Programme on Chemical Safety

^e^Krug et al. (2013b)

^f^Wei et al. (2014)

^g^Pili et al. (2012)

^h^Bauer et al. (2014)

^i^ http://pubchem.ncbi.nlm.nih.gov/compound/16682730#section=CLP-Hazard-Class-and-Category-Codes

^j^Pichichero et al. (2008)

^k^Zimmer et al. (2014)

The analyzed test compound panel includes also five environmental pollutants. For three of them, PBDE-99, PCB-153, and triadimefon, evidence of neurotoxicity is available. Indeed, the dual luminescence reporter assay identified all three compounds as potentially neurotoxic (Fig. 5d, Fig. S6B,C). PBDE-99 is a flame retardant containing polybrominated diphenyl ethers (PBDEs). It easily leaches out from furniture for example, and residues have been identified in house dust and food (Frederiksen et al. 2009), as well as in human blood, adipose and placental tissues, and breast milk (Costa et al. 2008; Furst 2006; Pellacani et al. 2014). Exposure to PBDEs has been associated with developmental neurotoxicity, endocrine dysfunction, and reproductive disorders (Costa et al. 2008; Darnerud 2008; Eskenazi et al. 2013; Gascon et al. 2012). In our assay, PBDE-99 acted predominantly on neurons with moderate cell number decrease, but had a more marked effect on the general promoter activity (Fig. 5d). Human exposure concentrations are in the range of 5–100 pM (Zimmer et al. 2014), which is below the active concentrations in the dual luminescence reporter assay seen in the present study. Future research should investigate whether long term exposure will lead to an in vitro toxicity signal at lower concentrations, or whether the effect of PBDE-99 depends on co-exposure together with other congeners (Eskenazi et al. 2013; Gascon et al. 2012).

Triadimefon is largely used in agriculture as a pesticide. Studies of acute effects in rodents have indicated a potential to induce neurobehavioral effects (Crofton 1996; Reeves et al. 2004), and toxicity to the neural crest (Zimmer et al. 2012). To our knowledge, no evidence for human direct neurotoxicity is available. In the dual luminescence reporter assay, triadimefon caused only relatively weak responses (Fig. S6B) suggesting that it does not rank among the most critical compounds for murine central neurons and ESCs at the tested concentrations. Polychlorinated biphenyls (PCBs) are food contaminants widely known for their potential carcinogenic and non-carcinogenic effects (http://www.epa.gov/epawaste/hazard/tsd/pcbs/pubs/effects.htm). In particular, a link between prenatal and postnatal exposure to PCBs and childhood cognitive function has been reported (Grandjean and Landrigan 2006; Jacobson and Jacobson 2001). In our study, the effects of PCB-153 were limited to neurons where it decreased the neural and general promoter activities at the highest concentration (Fig. S6C) without affecting the number of cells.

Methoxyacetic acid is the active metabolite of the widely used industrial chemical ethylene glycol monomethyl ether (Henley and Korach 2010). This compound may have some developmental toxicity in humans (Welsch 2005). In the dual luminescence reporter assay, methoxyacetic acid showed only a relatively weak signal. At concentrations of 50 µM, an approximately 40 % inhibition of neuronal promoter activity was seen in neurons, whereas an increase of neural promoter activity was obtained in ESCs (Fig. S6A). Given the relatively high blood concentrations of methoxyacetic acid estimated in humans exposed to the compound (Welsch 2005), our results showing statistically significant effects on the neural promoter at 50 µM should be considered as a positive signal.

The antifungal agent cyproconazole is a triazole like triadimefon. It is most likely devoid of neurotoxicity (http://www.fao.org/fileadmin/templates/agphome/documents/Pests_Pesticides/JMPR/Report10/Cyproconazole.pdf) and did not give a signal in our assay system (Fig. S6D).

We also analyzed the effects of five polypeptides: erythropoietin, neuregulin, G-CSF, IFN-β and oxytocin, testing concentrations in the range of reported in vivo concentrations (Table 2). Oxytocin did not have any effects in the dual luminescence reporter assay (Fig. S8A). In contrast, the four other compounds selectively increased general promoter activity in neurons (Fig. S8B,C,D and Fig. S9D). Also, the compounds did not affect ESCs. This is reminiscent of previous observations studying neurogenic polypeptides whose simultaneous activation of the neural and the general promoter has been described as a signature profile for growth factor-like activity (Xu et al. 2014). The absence of signs of neurotoxicity (i.e., decreased reporter activity) for these polypeptides seems plausible, given their essential role for neuronal development during embryogenesis and to neuronal function in the adult brain (Laske et al. 2009; Roysommuti et al. 2003).

Changes induced by all tested compounds on the three analyzed parameters in ESCs and in neurons are summarized in Table 2. The numbers given in column 3-8 correspond to the lowest observed adverse effect level (LOAEL), i.e., the lowest tested concentrations that caused a statistically significant decrease in a given parameter. Column 9 gives information about known concentrations that were found in humans. Column 10 gives the number of parameters changed for a given compound. The highest possible number is 4, corresponding to the promoter activities of the two different promoters in two cell types (ESCs and neurons). A change of reporter activity in the non-cytotoxic range, as scored here, was defined as a statistically significant decrease in reporter activity without a corresponding change in PI values. Non-neurotoxic controls generally had no impact on our test systems with the exception of propranolol that caused some minor changes at highest concentrations. An interesting exception to the rule that Tα1 promoter is most sensitive to neurotoxic insult is the impact of trimethyltin. Indeed, in neurons, the EF1α promoter LOAEL was at 50 nM, as compared to a LOAEL of 5 µM for Tα1 and PI. This demonstrates that for some type of toxicants the perturbance of cellular gene expression by a well-defined neurotoxicant is more readily detected by a general promoter driving protein translation (EF1α), than by the promoter driving expression of a neural-specific gene (Tα1). It clearly provides a justification for our dual promoter approach. Note also that in the analysis provided in this table increases in promoter activity were not included. Although these increases might be of interest for understanding mechanisms of activity of certain compounds, they did not seem to improve detection of neurotoxicants.

### Alert plots of neurotoxicity

**Fig. 6:**
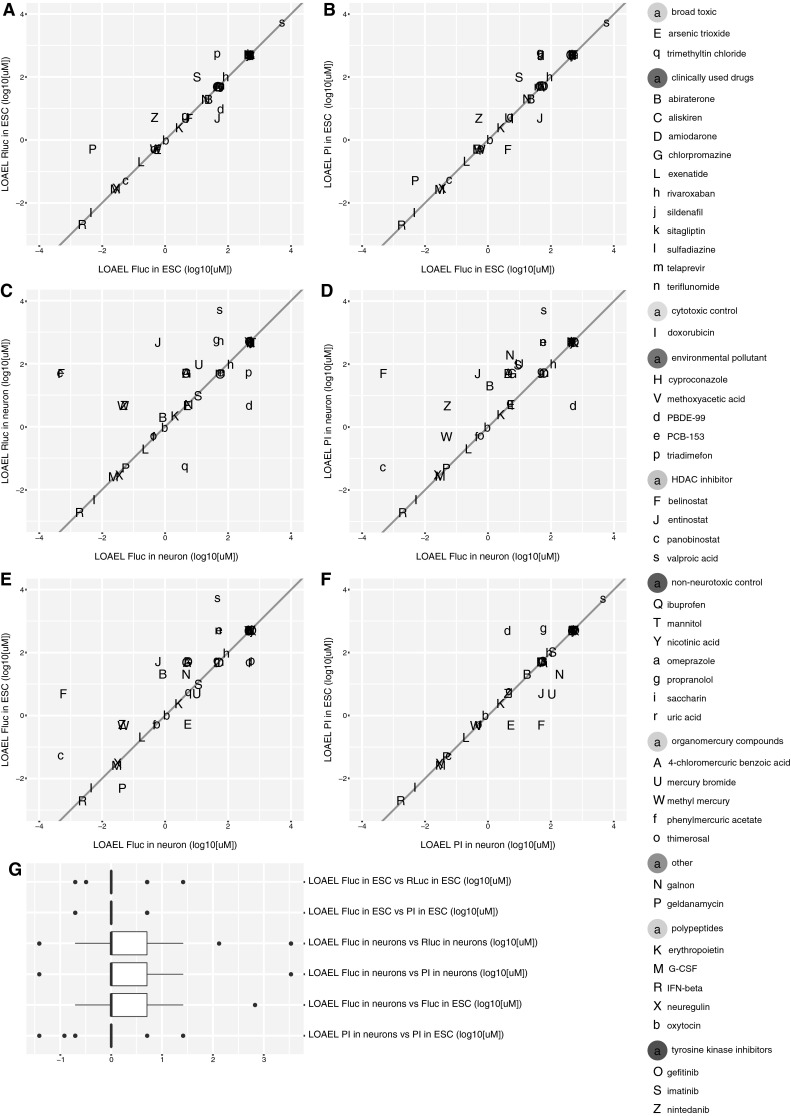
Direct comparison of LOAELs in alert plots. **a**–**f** A scatterplot of log10 transformed lowest adverse effect levels (LOAEL). **g** The *box plot* displays the distribution of distances perpendicular to the diagonal for the comparison of endpoint sensitivity of compounds. Distances of points below the diagonal are shown as negative values. Data are as in **a**–**f**

To obtain an overview over the effects induced by the test compounds, the lowest observed adverse effect concentrations (LOAEL) affecting the activities of the neuronal (FLuc) and general (RLuc) promoter were summarized in alert plots (Fig. 6). Alert plots were established separately for ESCs (Fig. 6a, b) and the derived neuronal cells (Fig. 6c, d). Analysis of ESCs resulted in a high correlation of FLuc and RLuc with data points close to the diagonal, indicating similar responses of the neuronal and the general promoter activities (Fig. 6a). A different scenario was obtained in the alert plot of the ESC-derived neuronal cells, where several compounds clustered to the upper left of the diagonal (Fig. 6c), indicating that these compounds affect the neuronal promoter at lower concentrations than the general promoter. Interestingly, well-known neurotoxicants as well as DNT compounds cluster in the upper left region, such as valproic acid and other HDAC inhibitors, and also methylmercury. Therefore, clustering of an unknown test compound to this region should be considered as a neurotoxic alert that requires further assessment. It is also of interest that for many compounds, toxicity was evident in neurons differentiated from ESCs, but not in non-differentiated ESCs. In a similar way, alert plots were set for FLuc and PI to study the relationship between neuronal promoter activation and cytotoxicity (Fig. 6b, d). Clustering to the upper left in these plots means that neuronal promoter inhibition occurs at lower concentrations than cytotoxicity. A direct comparison between LOAELs in ESCs and the derived neuronal cells was analyzed for PI (Fig. 6f) and FLuc (Fig. 6e). For PI, the measure of cytotoxicity, relatively similar results were obtained for both ESCs and neurons (Fig. 6f). In contrast, the responses for the neural promoter activity cluster to the lower right, indicating a higher sensitivity for neuronal cells compared to ESCs (Fig. 6e). Again, this increase in susceptibility of neuronal cells compared to their precursor cells was particularly pronounced for HDAC inhibitors. These alert plots of LOAELs obtained by the dual luminescence reporter assay will be helpful to obtain an overview of possible neurotoxic alerts of novel test compounds, especially since their scattering positions can now be compared to those of a number of positive and negative reference compounds.

Finally, an interesting feature of the here established dual fluorescence reporter assay is that compounds cannot only lead to a decrease in reporter gene expression, but also to an increase, or to biphasic curves where an increase is followed by a decrease. This was particularly evident for HDAC inhibitors, as well as for teriflunomide. Bimodal curves were obtained in ESCs, but a decrease of reporter activities was observed in neurons. To see whether these patterns of reporter gene expression were linked to known neurotoxicity, we analyzed the type of curve that was observed in different groups of compounds (Fig. S14). All tested clinically used compounds did not cause any positive effects in the dual luminescence reporter assay at concentrations known to occur in the blood of patients.

### PBPK modeling of hit compounds

**Fig. 7:**
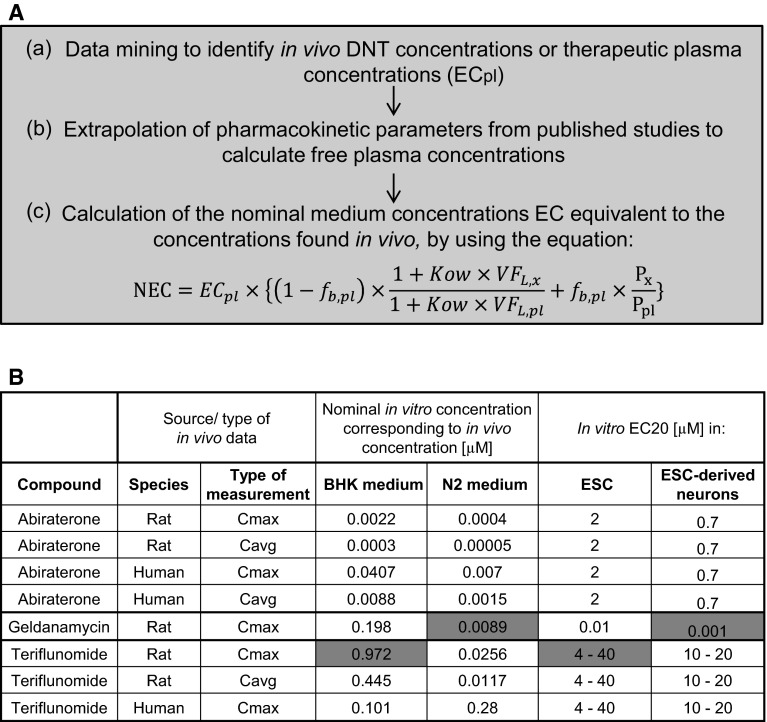
Reverse modeling of relevant in vivo plasma concentrations for comparison with toxic in vitro concentrations. **a** Outline of the workflow to determine the nominal effective concentration (NEC) in cell culture media. **b** Synopsis of NEC for the two media used in the study and the corresponding in vitro toxicity data (expressed as EC20) for selected compounds. NEC in embryonic pluripotent stem cells (ESCs) were compared to predicted concentrations in BHK medium; NEC in neurons were compared to predicted concentrations in N2 medium. Fields marked in *green* show effects in a similar concentration range (within factor 10) in in vitro tests (here) and in vivo data (literature). *Abbreviations*. *NEC* nominal effective concentration, *fb,pl* plasma bound fraction, *Kow* octanol:water partition coefficient, *VFL* volume fractions of lipids, *P* albumin concentration (or total protein concentration); suffix “*pl*” plasma; suffix “*x*” type of medium used in this study (color figure online)

To compare the effective/toxic concentrations found in our study with relevant in vivo concentrations, we used a reverse pharmacokinetic modeling approach for three exemplary compounds: teriflunomide, geldanamycin, and abiraterone. Firstly, we used a literature data mining approach to identify toxic or clinically relevant plasma concentrations reported in published animal studies (in relation to developmental toxicity or neurotoxicity) or clinical measurements (mostly in relation to drug efficacy). Then those values were used as points-of-departure to calculate the nominal equivalent concentrations (NEC) in both cell culture media used in our study, the BHK and N2 medium. These two media (used to expose the ESCs and the ESC-derived neurons) contained different amounts of protein and lipid and therefore required separate calculations of the NEC. The strategy to calculate the NEC was based on the assumption that only the free fraction of the drug (not bound to protein or lipid) is responsible for toxicity or pharmacological efficacy. Thus, free concentrations should be compared across systems, and for this it was important to calculate the NEC that would yield the same free concentration. This mathematical background is best exemplified by an example: if a drug D has clinical effects at a plasma concentration of 5 µM, and the free fraction is 10 % in human plasma, then experimental test systems should show an effect at a free drug concentration of 0.5 µM, if they react in the same way as humans. The NEC is the nominal concentration in a given test system that produces this free drug concentration. For instance, in test system A, there may be no protein or lipid in the medium. In this case, the NEC would be equal to the free concentration, i.e., 0.5 µM. In test system B, the free drug concentration may be only 1 % (due to high protein content). In this system, the NEC would be 50 µM (to obtain a free concentration of 0.5 µM). Accordingly, the different NEC were calculated for the two test systems (media) used here (Fig. 7a). For the calculation of the NEC, the parameters of lipid content (VFL) and albumin concentration (P) (or total protein concentration) were determined for each model from the literature or supplier data sheet information (Fig. S2A). The compound-specific values of plasma bound fraction and octanol:water partition coefficient were extracted from the literature to allow NEC calculation.

After the determination of the NEC, the next step of our strategy was to compare the NEC with the NOAEL for toxicity in our test systems. This procedure, and the conclusions therefrom, is detailed for the three model compounds below.

Abiraterone: a developmental toxicity study performed in rats by the Australian regulatory body for therapeutic goods, TGA (TGA 2014) and data from clinical measurements on the pharmacokinetics of abiraterone in healthy subjects (Goldberg and Berrios-Colon 2013) were used to identify relevant drug plasma concentrations. The average concentrations (Cavg) were calculated from the maximal concentration (Cmax), based on published pharmacokinetics studies. The NEC equation was finally applied as described in Materials and methods. The NEC of abiraterone in N2 medium was 0.05–0.4 nM when modeled from the rat DNT exposure, and 1.5–7 nM when human exposure data were used. The NEC in BHK medium (ECy) was 0.3–2.2 nM (for DT dose exposure) and 8.8–41 nM when calculated using the clinical dose. The LOAEL of abiraterone was 0.7–2 µM in neurons/ESCs. Comparison of the LOAEL and NEC shows a difference of 50–100 fold, and thus suggests that the clinical drug concentrations are far below the one causing effects in our in vitro test. Moreover, the effects observed in the rat developmental toxicity study occurred at lower concentration than the ones required to affect neurodifferentiation in our neuronal model (Fig. 7b, Fig. S13).

Geldanamycin: Data on the developmental toxicity of geldanamycin in vivo were not found, but effects on neurodifferentiation were observed in rats by Sun et al. (2012) at a dose of 0.2 mg/kg/day. Extrapolation of pharmacokinetics data from Supko et al. (Supko et al. 1995) allowed then the calculation of the Cmax and of the respective NEC.

The NEC of geldanamycin was 8.9 nM in N2 medium and 198 nM in BHK medium when modeled from the rat study described by Sun et al. (2012). In our in vitro system, geldanamycin showed an EC20 of 1–10 nM. The effects of geldanamycin in our in vitro model thus closely reflected the in vivo concentration range (Fig. 7b, Fig. S13).

Finally, we compared the in vitro-in vivo toxicity/clinical data for teriflunomide. The developmental toxicity dose of the immunomodulatory drug was extrapolated from an in vivo study on the effect of teriflunomide on the offspring of drug-exposed rats (FDA 2012). Clinical pharmacokinetics information was extracted from a study on healthy individuals treated with the drug (TGA 2013). The NEC of teriflunomide in N2 medium was 12–26 nM when calculated from the rat data, and 280 nM when clinical plasma level data were used. The NEC of teriflunomide in BHK medium was 445–970 nM (for DT dose exposure) and 100 nM when calculated using the clinical doses. In our system, teriflunomide showed various effects in a concentration range of 4–40 µM. The NOAEL of teriflunomide in ESCs was in the same range (i.e., same order of magnitude) as the in vivo data, while the neuronal system was less sensitive to the drug (35 fold) (Fig. 7b, Fig. S13). These three examples indicated that NOAELs identified here may either be directly clinically relevant, be partially relevant (to create an alert), or be outside the realistic clinical range, and may therefore be neglected.

## Conclusion

In this study, a tool to identify test compounds with potential neurotoxic effects has been established. This high-throughput dual luminescence reporter assay provides a biologically relevant, embryonic stem cell-based testing system, which is particularly useful for the screening of high numbers of test compounds.

## Electronic supplementary material

Below is the link to the electronic supplementary material.

Supplementary material 1 (PDF 2927 kb)
