# Supplementary material for: Fingerprinting of neurotoxic compounds using a mouse embryonic stem cell dual luminescence reporter assay
Source: Arch Toxicol. 2016 Mar 25;91(1):365–91. doi: 10.1007/s00204-016-1690-2 (PMC5225183; doi:10.1007/s00204-016-1690-2)
Supplement: Supplementary file 1 — Supplementary material 1 (PDF 2927 kb) [file 204_2016_1690_MOESM1_ESM.pdf]

Supplemental Material

Fingerprinting of neurotoxic compounds using a mouse embryonic stem cell dual luminescence reporter assay

M. Colaianna, S. Ilmjärv, H. Peterson, I. Kern, S. Julien, M. Baquié, G. Pallocca, S. Bosgra, A. Sachinidis, J.G. Hengstler, M. Leist, KH Krause

Table of contents

• **Supplemental Material, Table S1**
Classification of compounds in three classes based on clinical evidence for neurotoxicity

• **Supplemental Material, Figure S1**
Type of response curves as a function of compound category.

• **Supplemental Material, Figure S2**
In vitro - in vivo comparison of toxicity data by PBPK modeling
Values for lipid fraction (VFL), albumin concentration (P) and total protein concentration (P)* in rat and human plasma and in N2 (x) and BHK (y) medium. Values for plasma bound fraction (fb,pl); octanol:water partition coefficient (Kow) and molecular weight (MW).

• **Supplemental Material, Figures S3 and S4**
Effect of well-known non-toxic compounds (negative controls) on neuronal (FLuc), general (RLuc) promoter activities and on DNA content (PI assay).

• **Supplemental Material, Figures from S5 to S12**
Effect of other compounds of ESNATS collection on neuronal (FLuc), general (RLuc) promoter activities and on DNA content (PI assay).

• **Supplemental Material, Figure S13**
PBPK modelling of hit compounds
Graphical representation of the relevant concentrations of hit compounds.

• **Supplemental Material, Figure S14**
A scatterplot of log10 transformed LOAEL and a principal component analysis (PCA) of compounds.

• **Supplemental Figure Legends**

• **Supplemental Bibliography**

Table S1

Classification of compounds in three classes based on clinical evidence for neurotoxicity

Non-neurotoxic (no evidence for DNT/NT)	Possibly (developmental) neurotoxic	Well-established (developmental) neurotoxic
Saccharin	Panobinostat*	Valproic acid[e]
Ibuprofen	Entinostat*	Methylmercury[f]
Omeprazole	Belinostat*	PBDE-99[g]
Nicotinic acid	Methoxyacetic acid*	PCB-153[h]
Uric acid	Phenylmercuric acetate**	Arsenic trioxide[i]
D-mannitol	4-chloromercuric benzoic acid**	Trimethyltin chloride[l]
Propranolol	Mercury bromide**	Chlorpromazine[m]
G-CSF	Thimerosal**	
Erythropoietin	Triadimefon***	
Neuregulin	Cyproconazole***	
Oxytocin	Geldanamycin***	
Sildenafil	Abiraterone***	
Rivaroxaban	IFN-beta***	
	Nintedanib [a]	
	Amiodarone [b]	
	Sulfadiazine [c]	
	Imatinib [d]	
	Gefitinib[n]	

*Belonging to HDACi family or similar mode of action as valproic acid, a known DNT toxicant (Jentink et al. 2010);

** Organo-mercury compounds, similar mode of action as methylmercury, known DNT/NT toxicant (Grandjean and Landrigan 2006)

*** Positive hit in other ESNATS test battery test systems (Zimmer et al. 2014)

[a] Patejdl et al. 2013

[b] Orr and Ahlskog 2009

[c] Reboli and Mandler 1992

[d] Rinne et al., 2012

[e] Krug et al. 2013

[f] Grandjean and Herz 2015

[g] Eskenazi et al. 2013

[h] Grandjean and Landrigan 2006

[i] Grandjean and Herz 2015

[l] Kreyberg et al. 1992

[m] Morris et al. 2009

[n] It is inhibiting signal cascades important in development, and it could therefor cause DNT. Most tyrosine kinase inhibitors are recommended not to be used during pregnancy. Some evidences of *in vivo* fetotoxicity for gefitinib were reported in a European public assessment report by the European Medicines Agency (EMA, 2008) and by Food and Drug Administration (FDA, 2015)

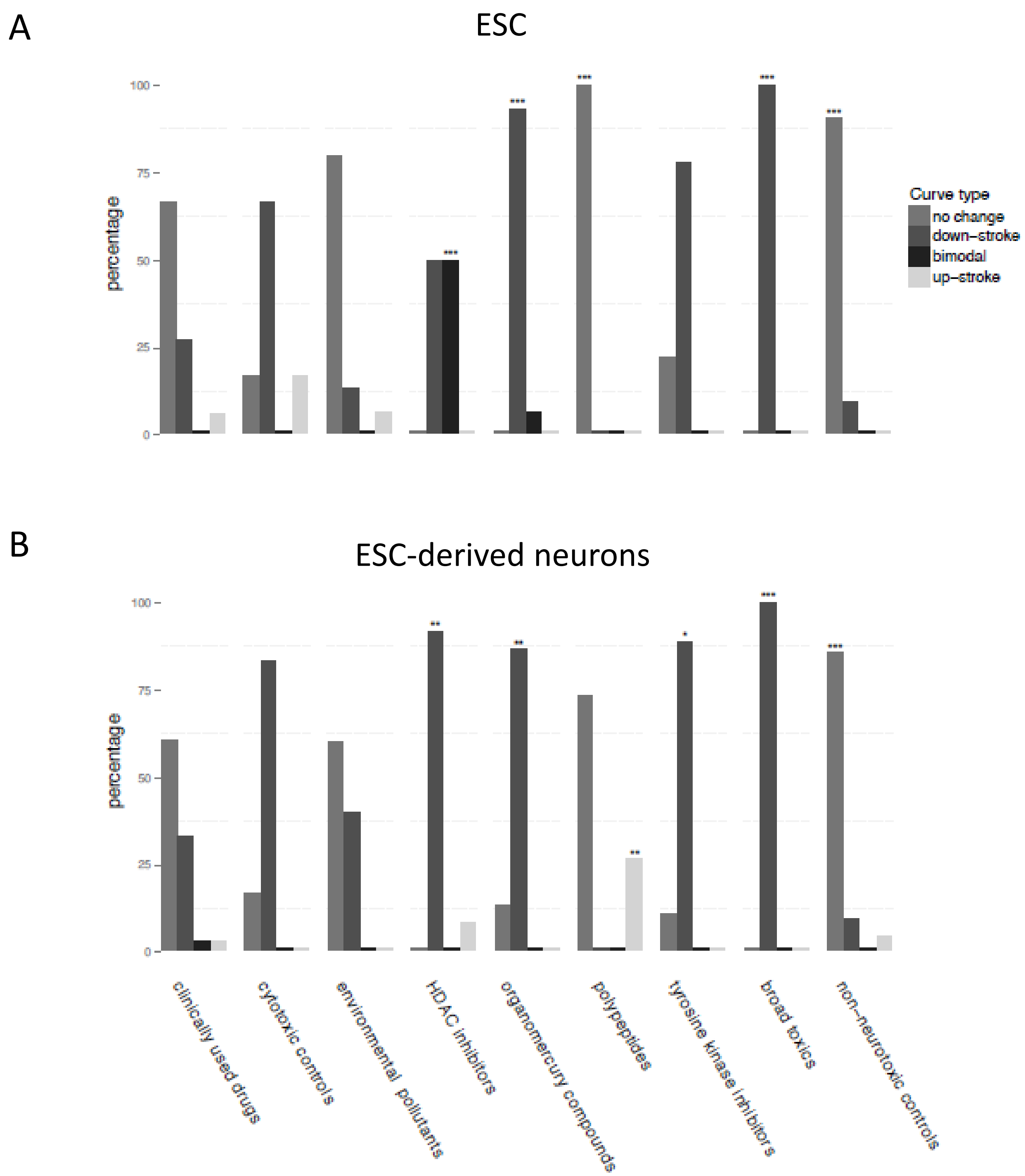

A
ESC
percentage
100
75
50
25
0
Curve type
no change
down–stroke
bimodal
up–stroke
B
ESC-derived neurons
percentage
100
75
50
25
0
clinically used drugs
cytotoxic controls
environmental pollutants
HDAC inhibitors
organomercury compounds
polypeptides
tyrosine kinase inhibitors
broad toxics
non–neurotoxic controls

Fig. S1

A	Rat plasma	Human plasma	N2 medium (x)	BHK medium (y)
Lipid fraction (VFL) [mg/l]	3600	6000	-	160
Albumin concentration (P) [µM]	421	600	-	36
Total protein concentration (P)* [µM]	877	1000	10.9	63

B	**Kow**	**fb,pl**	**MW**
**Abiraterone**	131826	0.998	349.5 g/mol

Concentrations in [µM]	**Rat**		**Human**	
	C_{max}	C_{avg}	C_{max}	C_{avg}
Total concentration *in vivo (ECpl)*	3.09E-02	4.01E-03	6.47E-01	1.40E-01
Free concentration *in vivo*	1.30E-07	1.68E-08	1.63E-06	3.53E-07
Equivalent total concentration *in vitro (ECx)*	3.83E-04	4.97E-05	7.04E-03	1.52E-03
Equivalent total concentration *in vitro (ECy)*	2.22E-03	2.88E-04	4.07E-02	8.80E-03

C	**Kow**	**fb,pl**	**MW**
**Geldanamycin**	417	0.990	560.7 g/mol

Concentrations in [µM]	**Rat**
	C_{max}
Total concentration *in vivo (ECpl)*	2.23E-00
Free concentration *in vivo*	8.91E-03
Equivalent total concentration *in vitro (ECx)*	8.91E-03
Equivalent total concentration *in vitro (ECy)*	1.98E-01

D	**Kow**	**fb,pl**	**MW**
**Teriflunomide**	327	0.995	270.2 g/mol

Concentrations in [µM]	**Rat**		**Human**
	C_{max}	C_{avg}	C_{avg}
Total concentration *in vivo (ECpl)*	1.11E+01	5.09E+00	1.65E+02
Free concentration *in vivo*	2.56E-02	1.17E-02	2.80E-01
Equivalent total concentration *in vitro (ECx)*	2.56E-02	1.17E-02	2.80E-01
Equivalent total concentration *in vitro (ECy)*	9.72E-01	4.45E-01	1.01E-01

Fig. S2

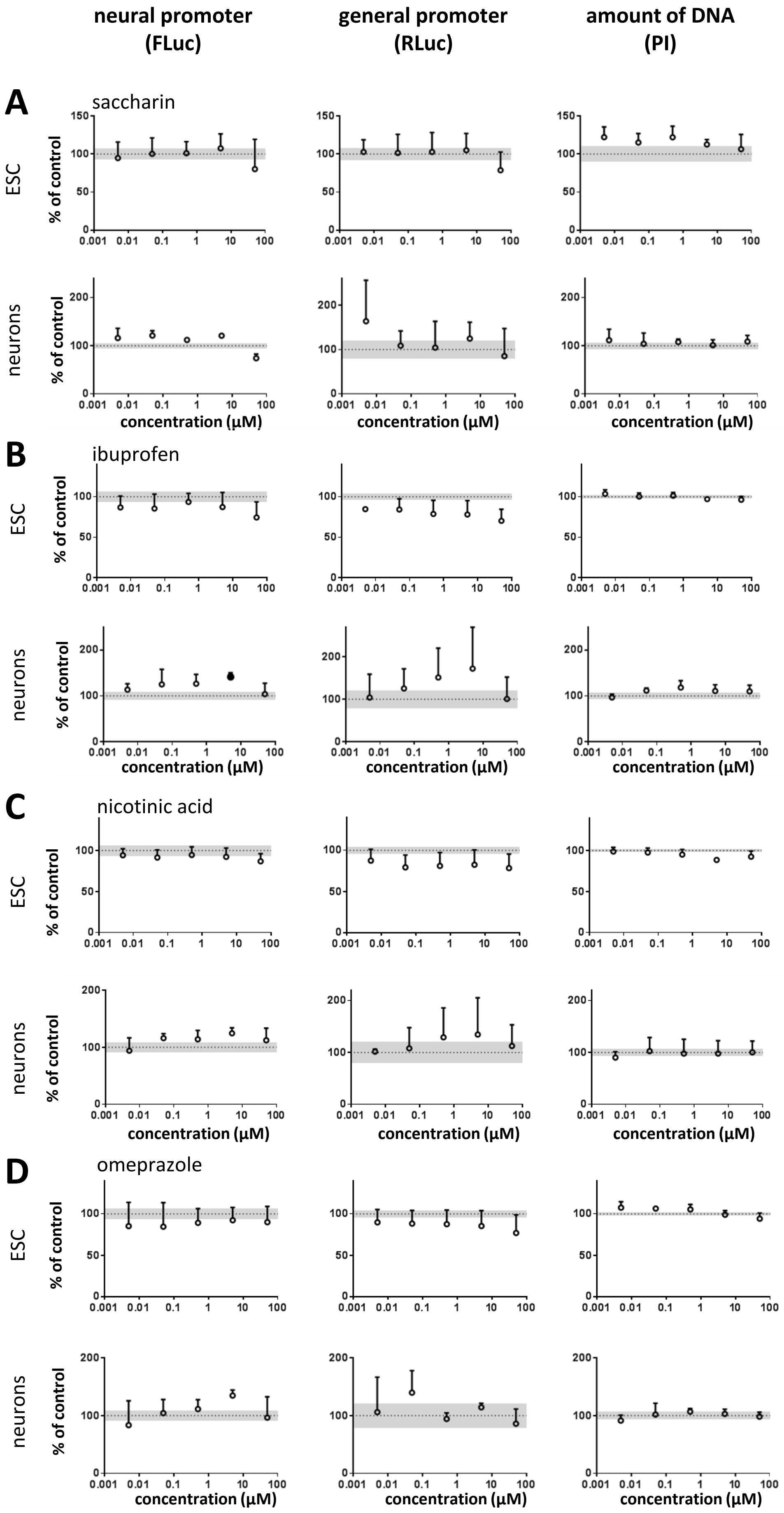

neural promoter (FLuc)
general promoter (RLuc)
amount of DNA (PI)
A
saccharin
B
ibuprofen
C
nicotinic acid
D
omeprazole
ESC
neurons
% of control
concentration (µM)

Fig. S3

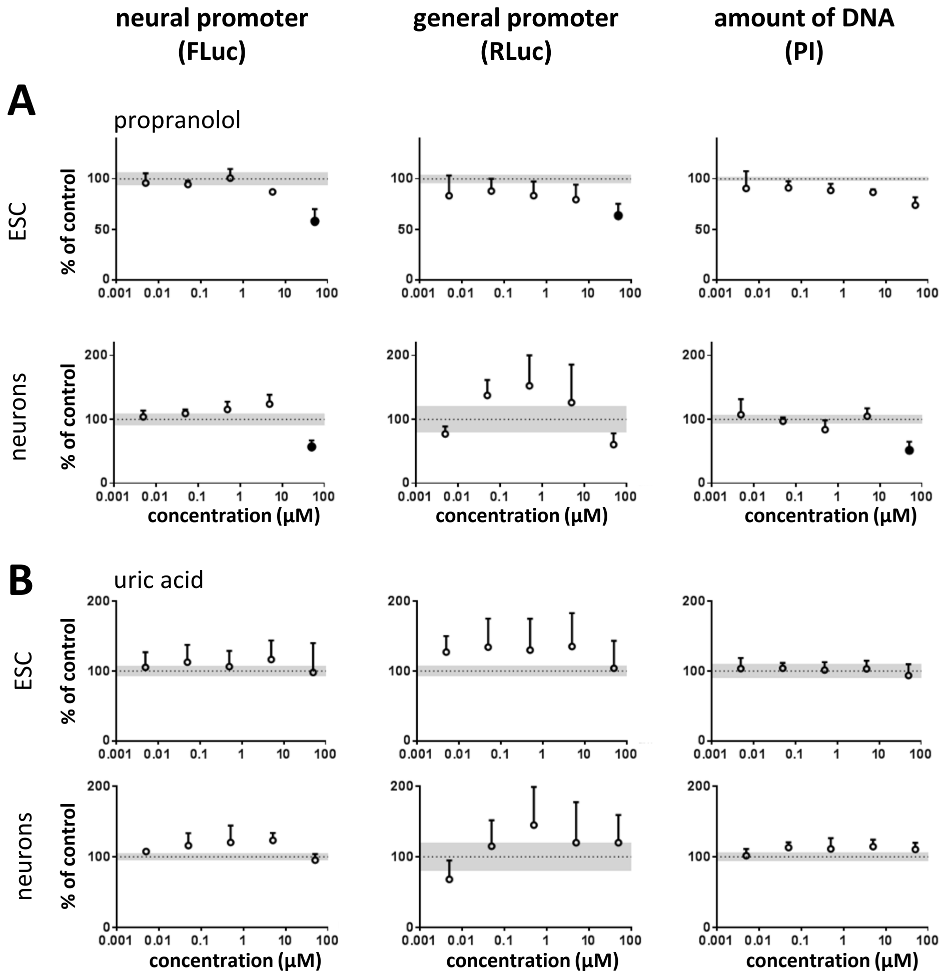

neural promoter (FLuc)
general promoter (RLuc)
amount of DNA (PI)
A
propranolol
ESC
neurons
% of control
concentration (µM)
B
uric acid
ESC
neurons
% of control
concentration (µM)

Fig. S4

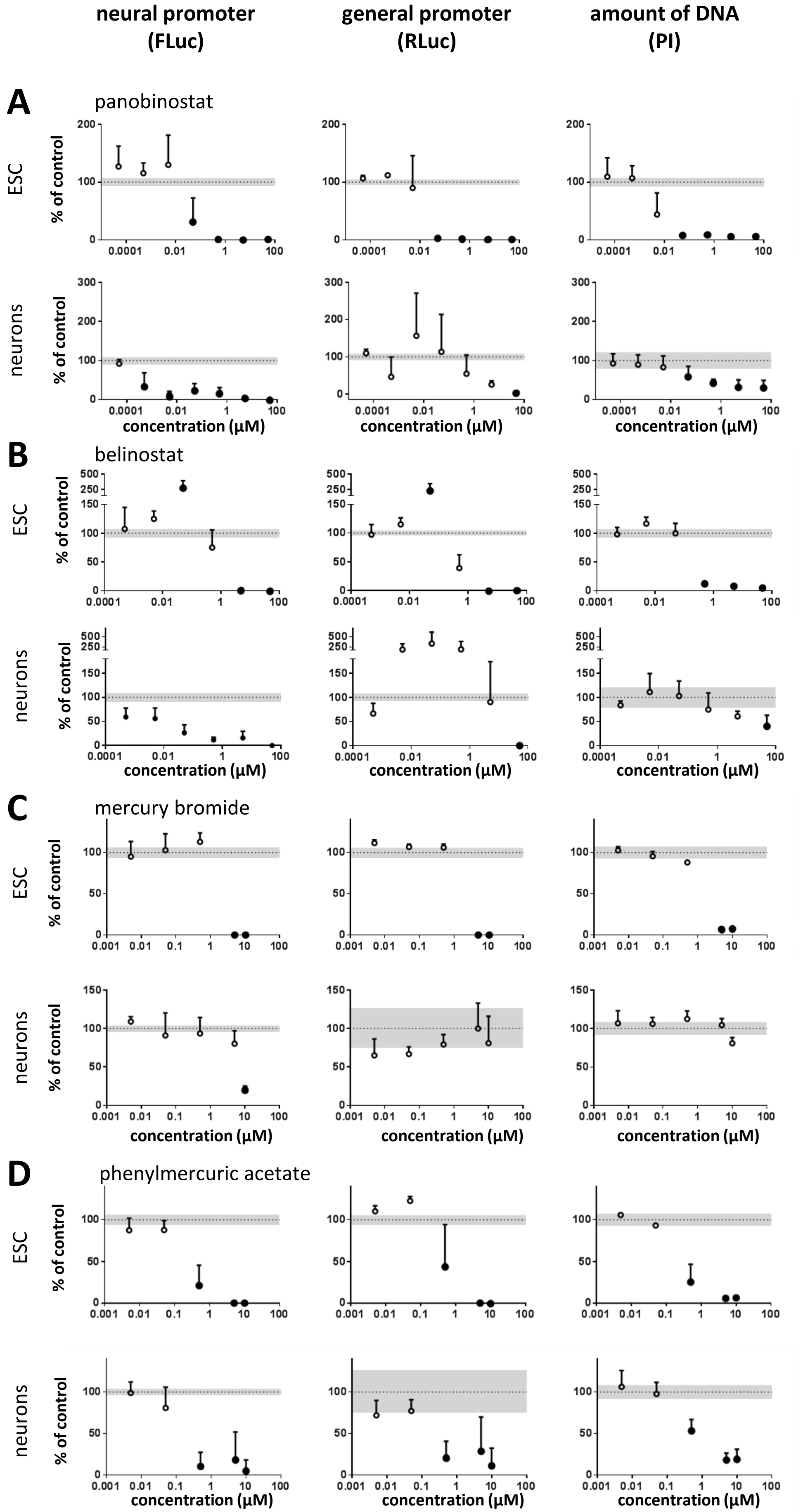

neural promoter (FLuc)
general promoter (RLuc)
amount of DNA (PI)
A
panobinostat
B
belinostat
C
mercury bromide
D
phenylmercuric acetate
ESC
neurons
% of control
concentration (µM)

Fig. S5

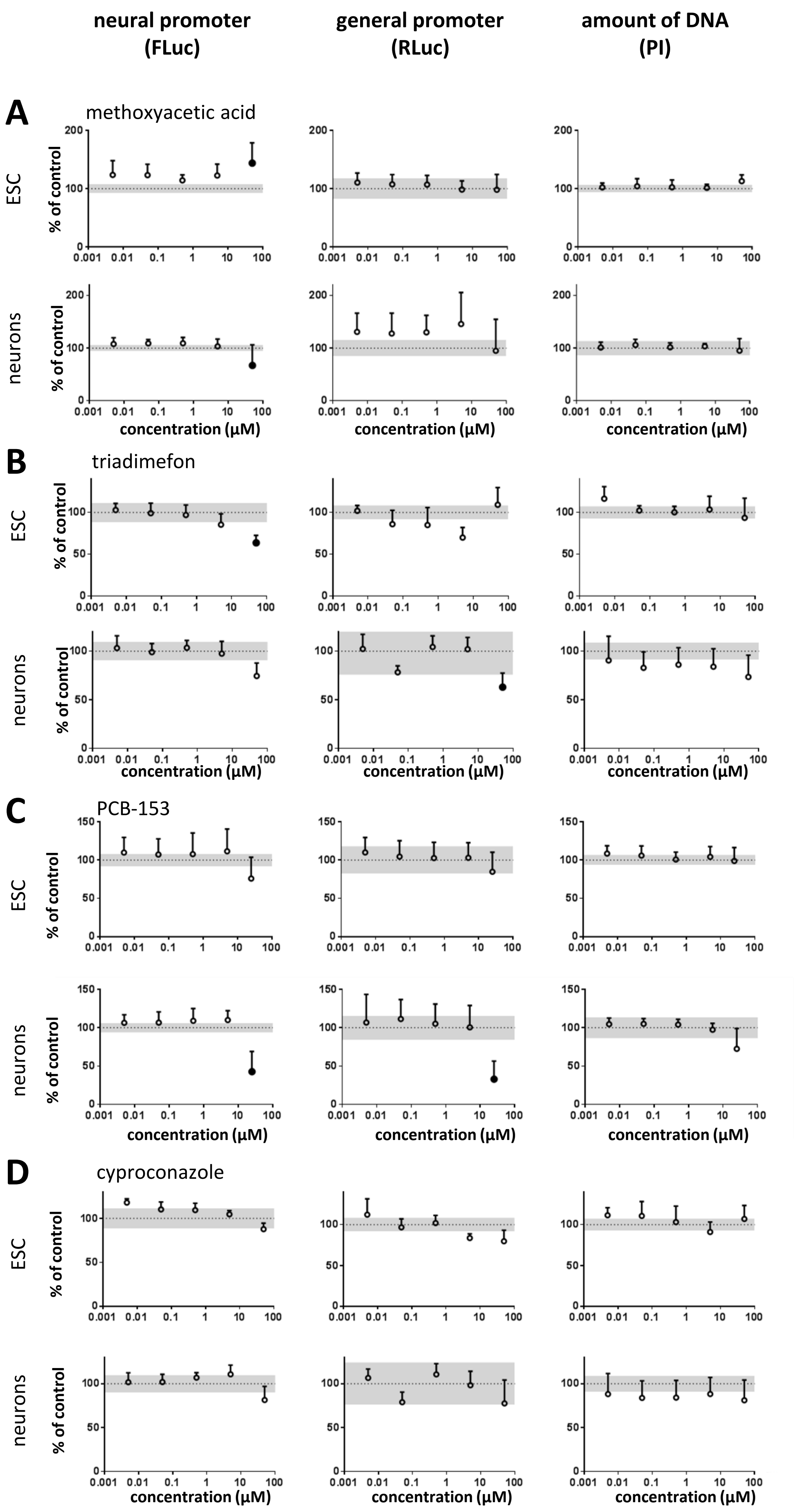
neural promoter (FLuc)
general promoter (RLuc)
amount of DNA (PI)
A
methoxyacetic acid
B
triadimefon
C
PCB-153
D
cyproconazole
ESC
neurons
% of control
concentration (µM)
0.001
0.01
0.1
1
10
100
0
50
100
150
200

Fig. S6

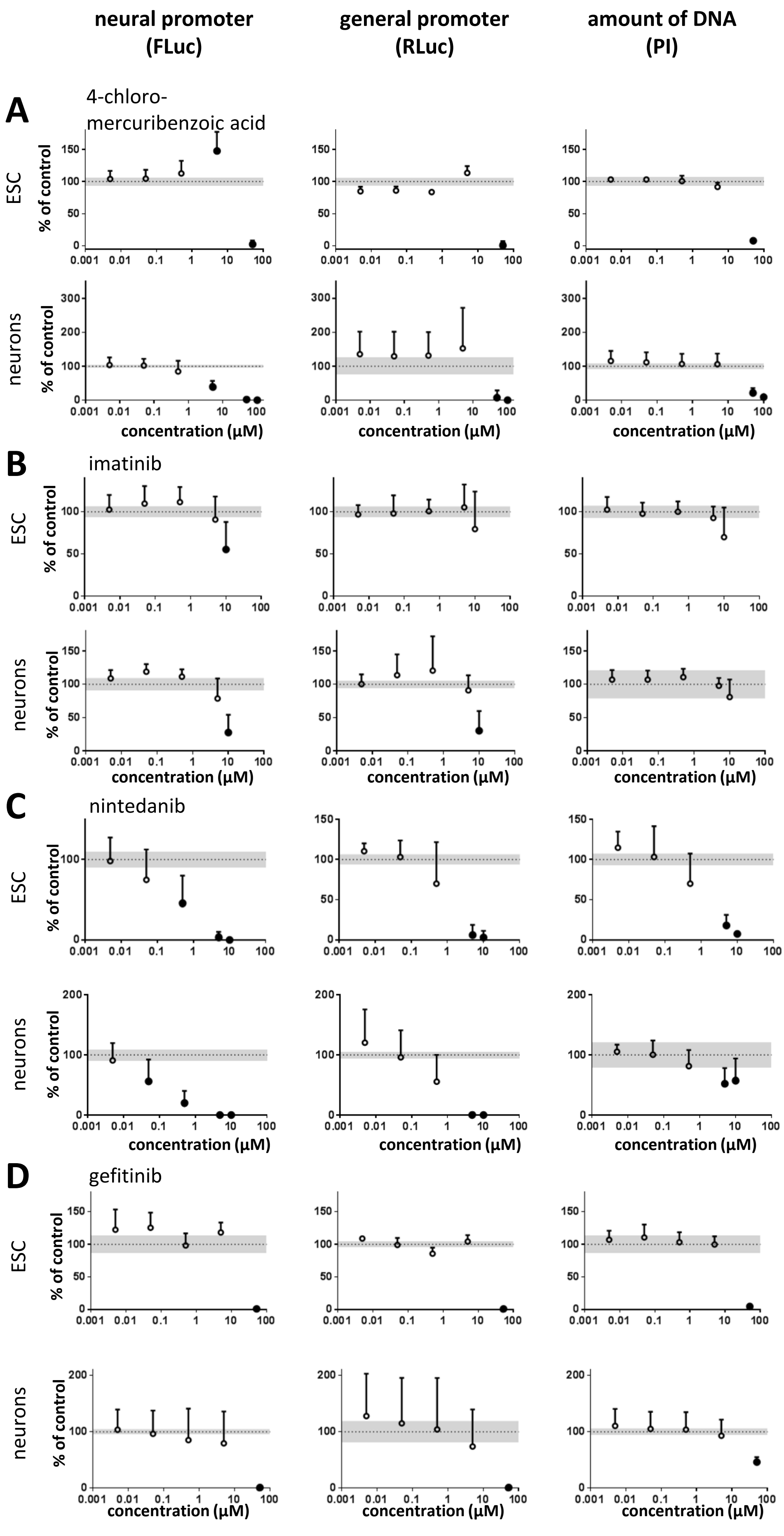
neural promoter (FLuc)
general promoter (RLuc)
amount of DNA (PI)
A
4-chloro-mercuribenzoic acid
B
imatinib
C
nintedanib
D
gefitinib
ESC
neurons
% of control
concentration (µM)

Fig. S7

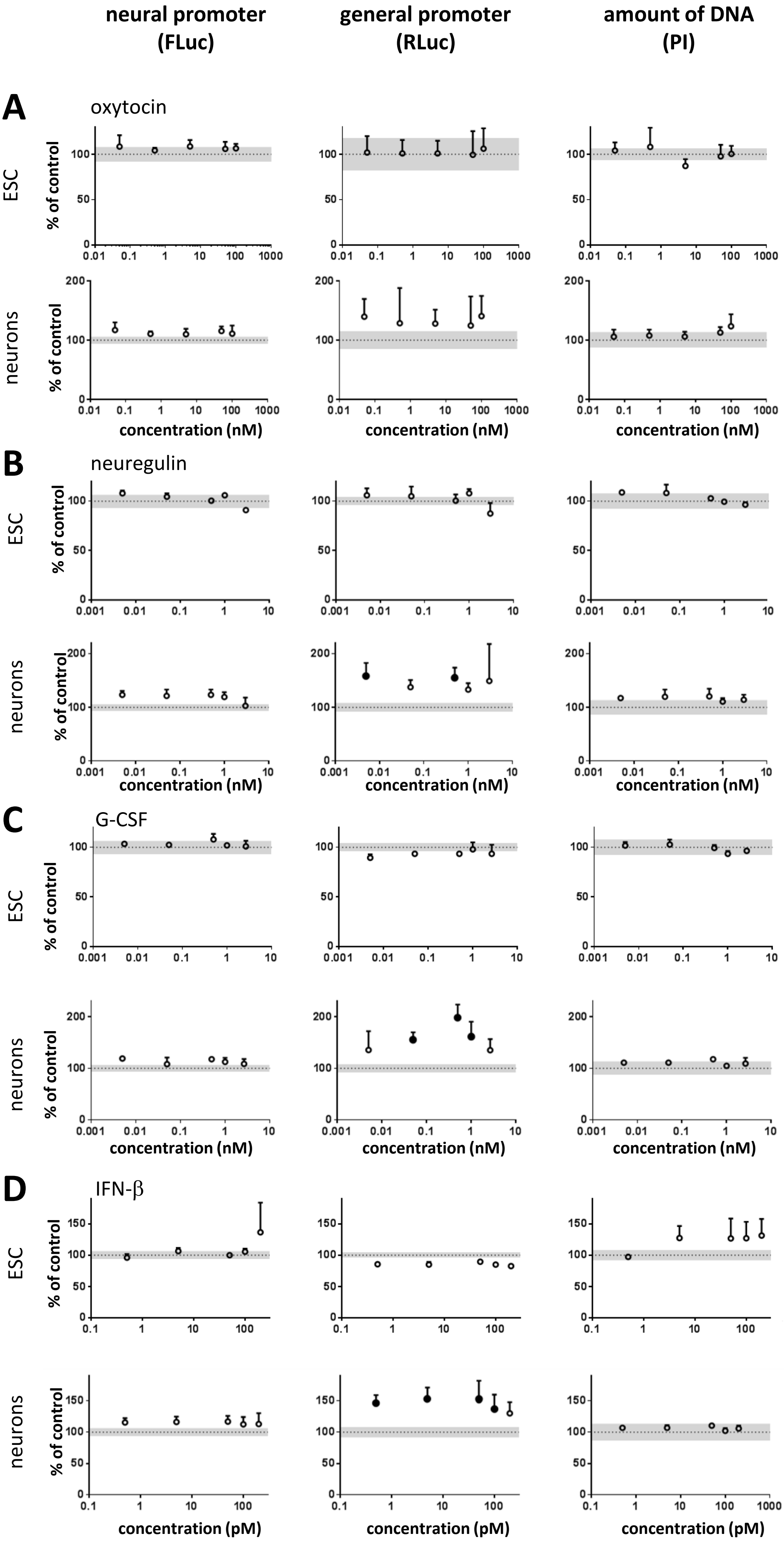
neural promoter (FLuc)
general promoter (RLuc)
amount of DNA (PI)
A
oxytocin
B
neuregulin
C
G-CSF
D
IFN-β
ESC
neurons
% of control
concentration (nM)
concentration (pM)

Fig. S8

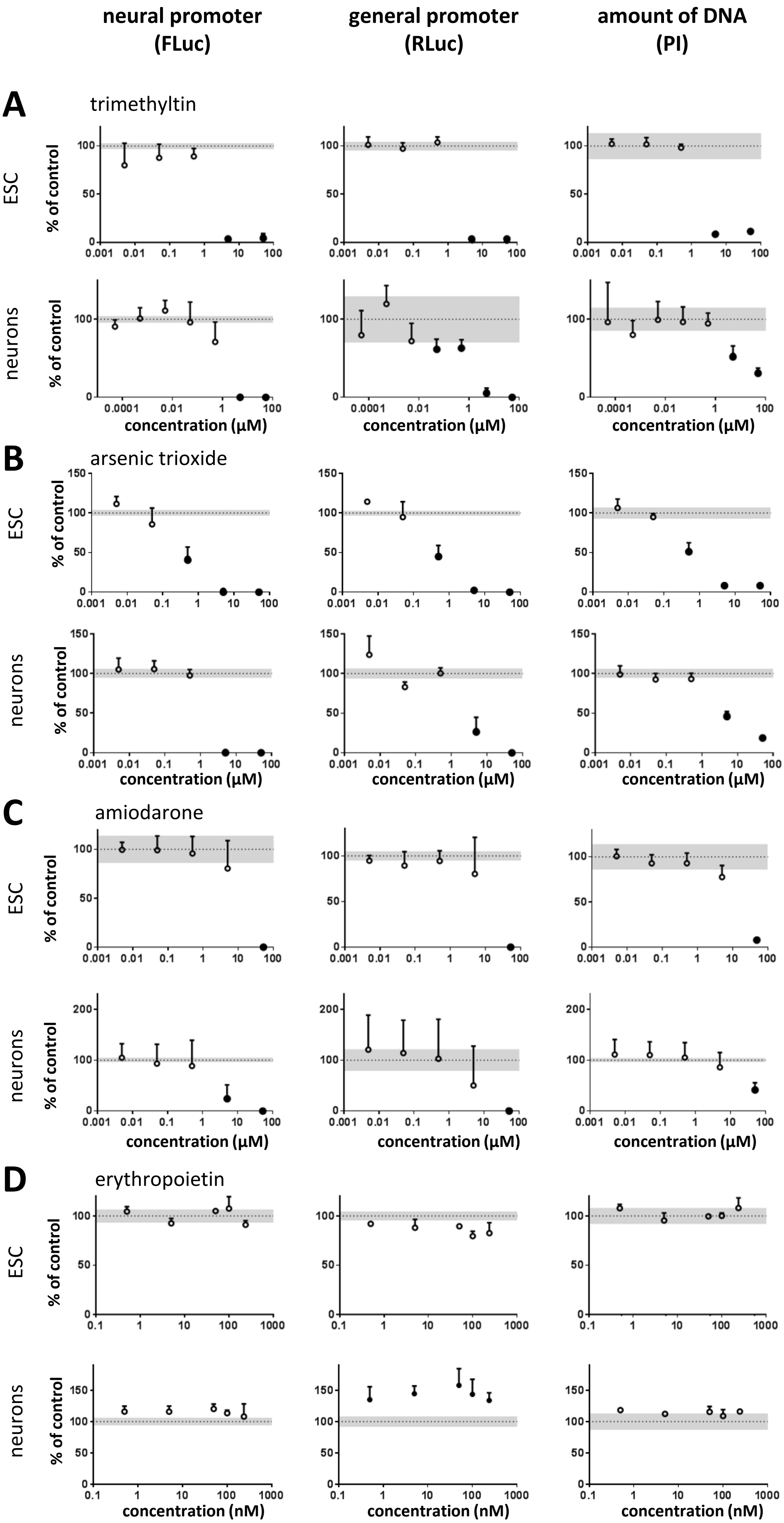
neural promoter (FLuc)
general promoter (RLuc)
amount of DNA (PI)
A
trimethyltin
B
arsenic trioxide
C
amiodarone
D
erythropoietin
ESC
neurons
% of control
concentration (µM)
concentration (nM)

Fig. S9

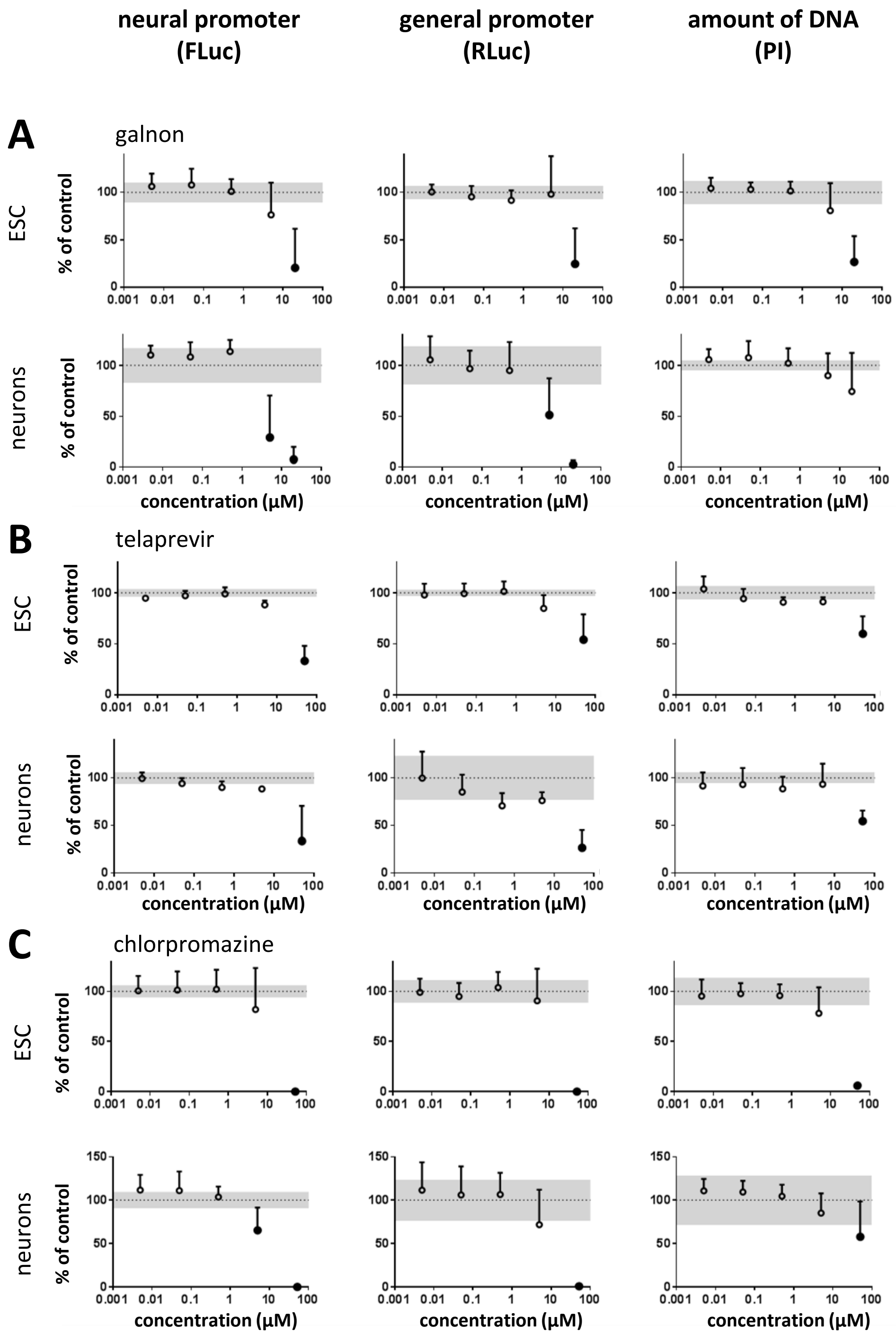

neural promoter (FLuc)
general promoter (RLuc)
amount of DNA (PI)
A
galnon
B
telaprevir
C
chlorpromazine
ESC
neurons
% of control
concentration (µM)
0.001 0.01 0.1 1 10 100
0 50 100 150

Fig. S10

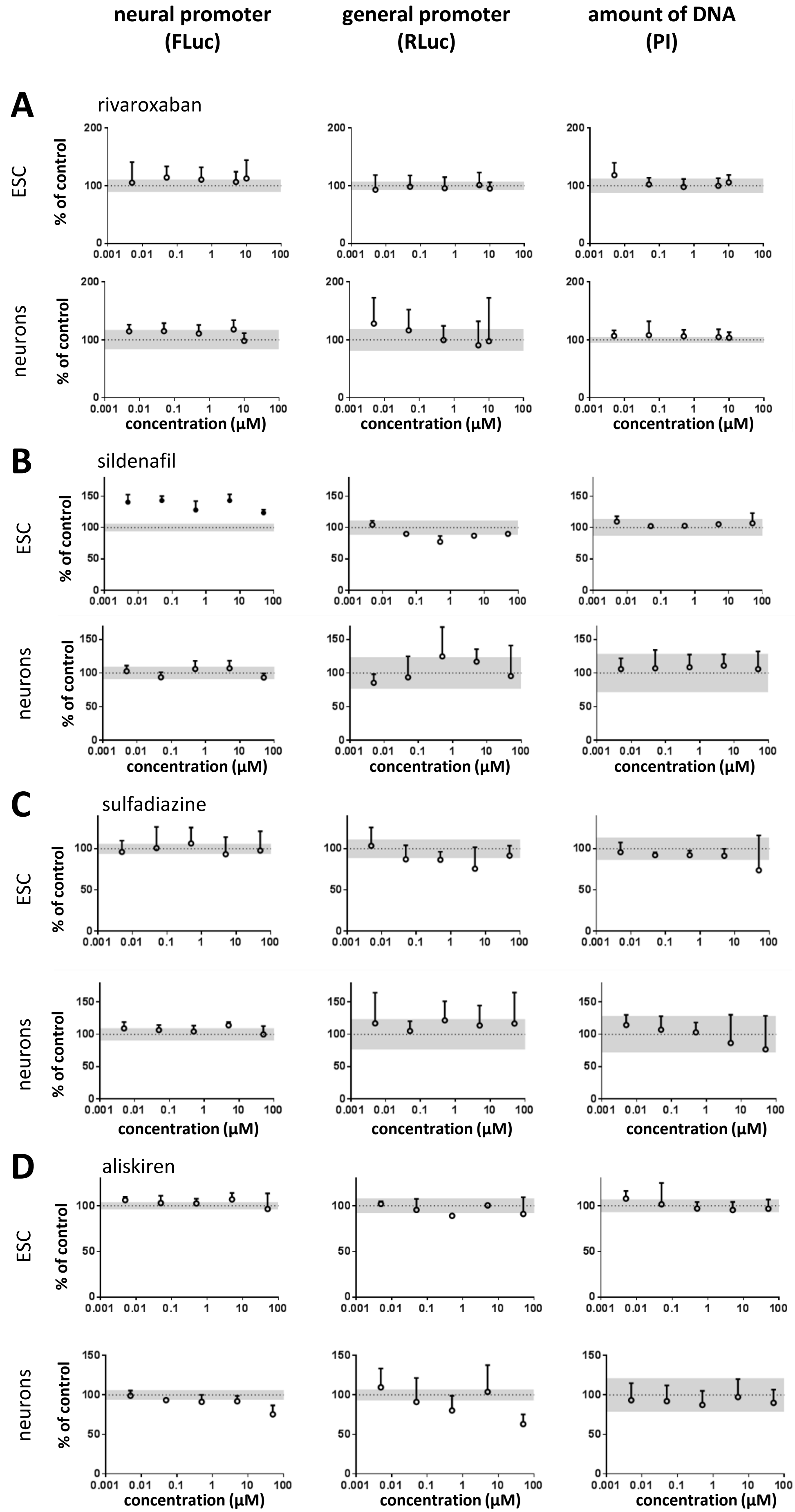
neural promoter (FLuc)
general promoter (RLuc)
amount of DNA (PI)
A
rivaroxaban
B
sildenafil
C
sulfadiazine
D
aliskiren
ESC
neurons
% of control
concentration (µM)

Fig. S11

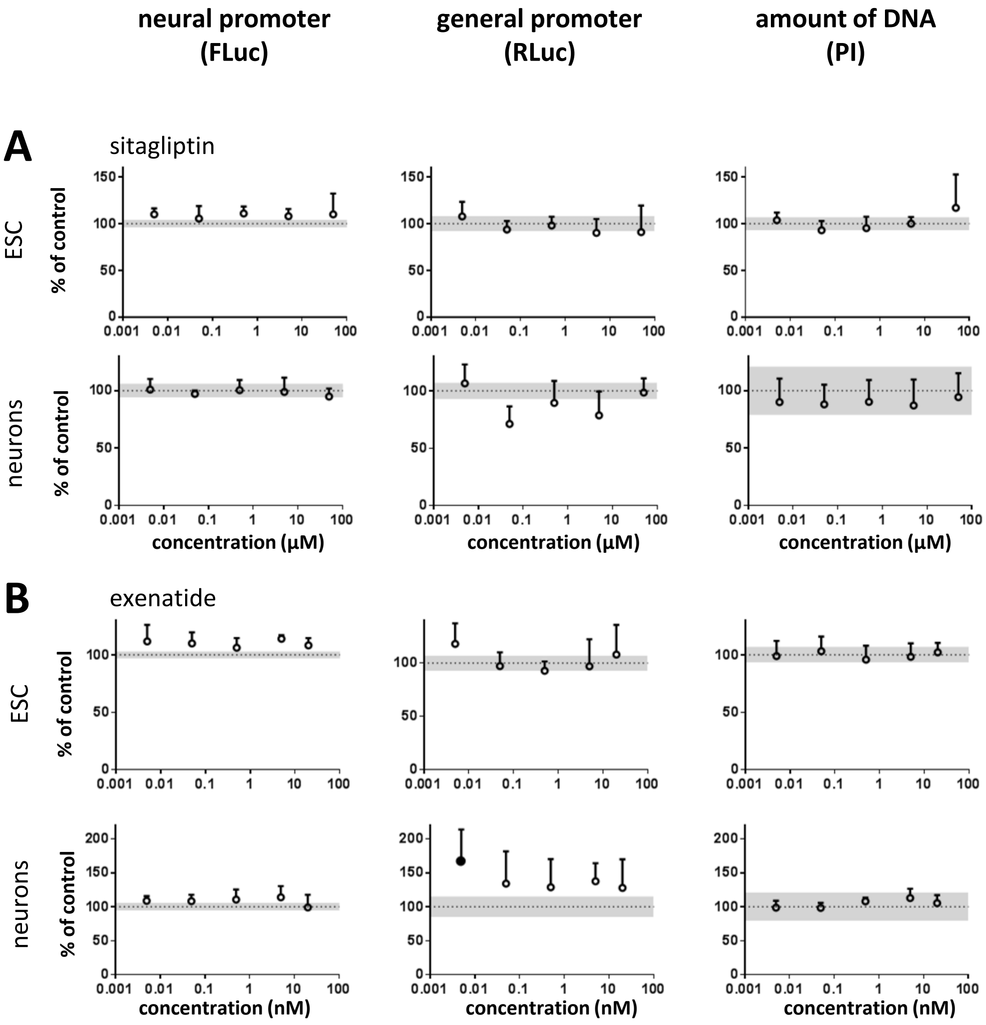
neural promoter (FLuc)
general promoter (RLuc)
amount of DNA (PI)
A
sitagliptin
ESC
neurons
% of control
concentration (µM)
B
exenatide
concentration (nM)
0.001
0.01
0.1
1
10
100
0
50
100
150
200

Fig. S12

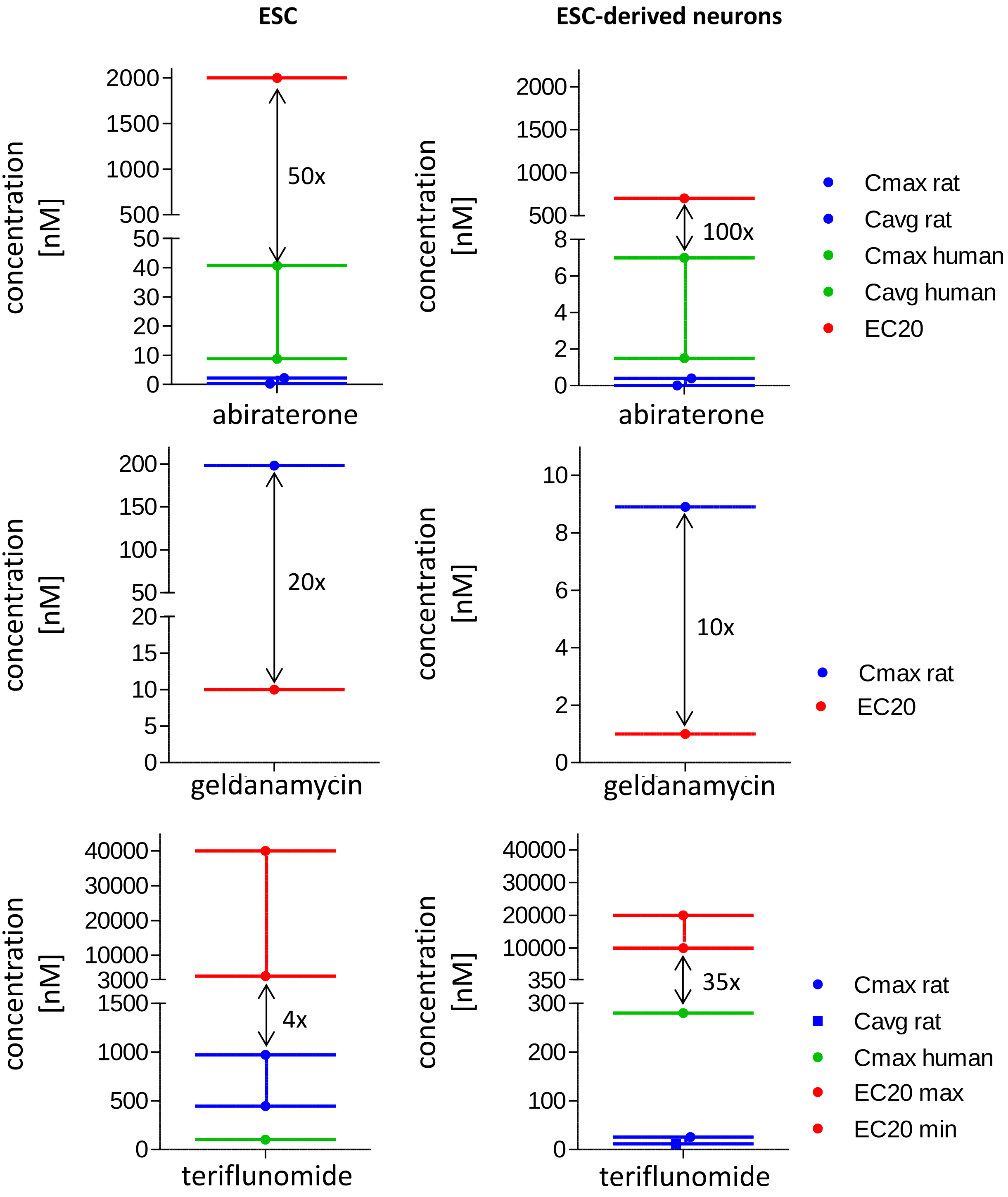

ESC
ESC-derived neurons
concentration [nM]
2000
1500
1000
500
50
40
30
20
10
0
50x
abiraterone
100x
8
6
4
2
Cmax rat
Cavg rat
Cmax human
Cavg human
EC20
200
150
100
15
5
20x
geldanamycin
10x
40000
30000
20000
10000
3000
4x
teriflunomide
350
300
35x
EC20 max
EC20 min

Fig. S13

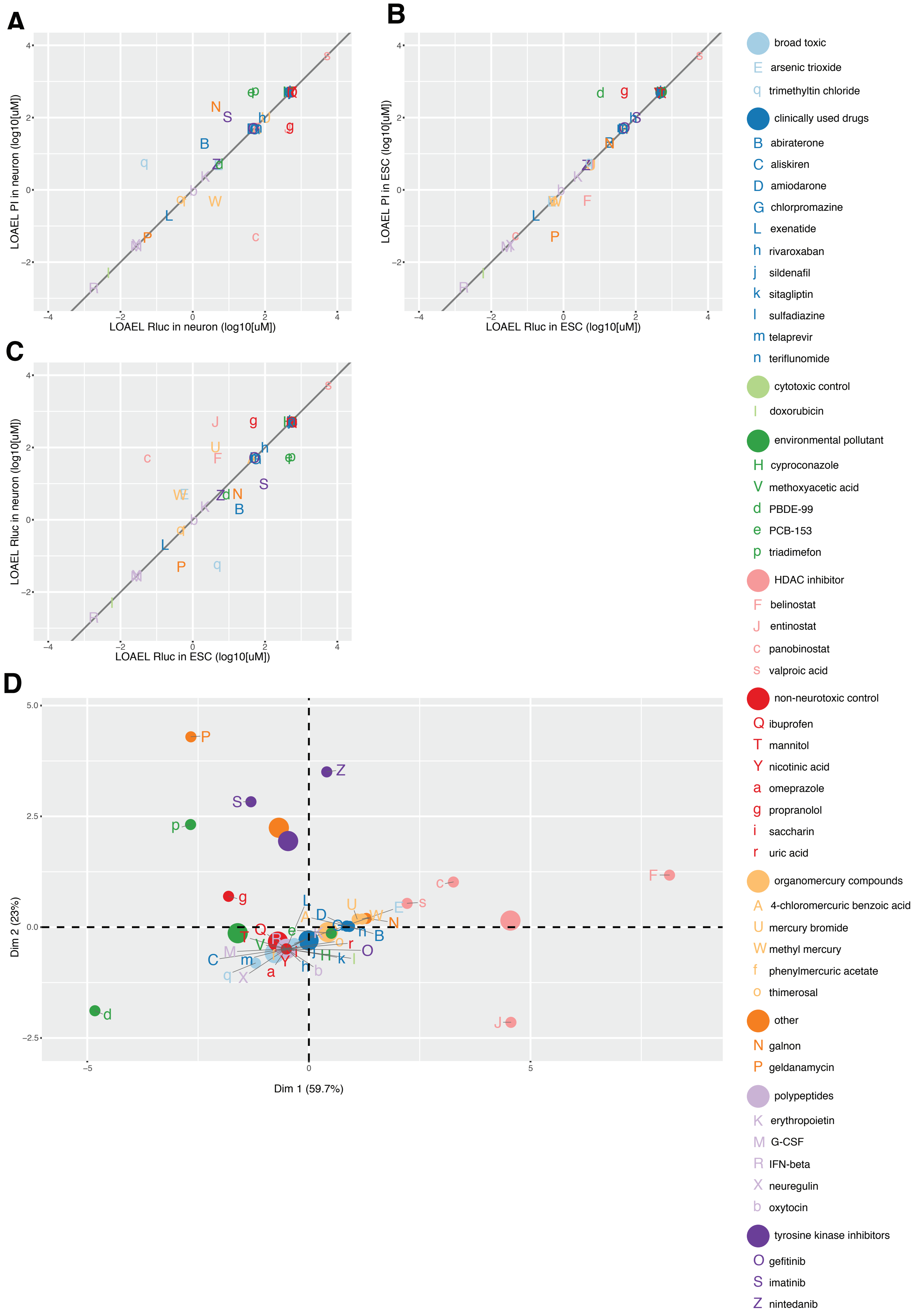

A
LOAEL PI in neuron (log10[uM])
LOAEL Rluc in neuron (log10[uM])
B
LOAEL PI in ESC (log10[uM])
LOAEL Rluc in ESC (log10[uM])
C
LOAEL Rluc in neuron (log10[uM])
LOAEL Rluc in ESC (log10[uM])
D
Dim 2 (23%)
Dim 1 (59.7%)
broad toxic
E arsenic trioxide
q trimethyltin chloride
clinically used drugs
B abiraterone
C aliskiren
D amiodarone
G chlorpromazine
L exenatide
h rivaroxaban
j sildenafil
k sitagliptin
l sulfadiazine
m telaprevir
n teriflunomide
cytotoxic control
I doxorubicin
environmental pollutant
H cyproconazole
V methoxyacetic acid
d PBDE-99
e PCB-153
p triadimefon
HDAC inhibitor
F belinostat
J entinostat
c panobinostat
s valproic acid
non-neurotoxic control
Q ibuprofen
T mannitol
Y nicotinic acid
a omeprazole
g propranolol
i saccharin
r uric acid
organomercury compounds
A 4-chloromercuric benzoic acid
U mercury bromide
W methyl mercury
f phenylmercuric acetate
o thimerosal
other
N galnon
P geldanamycin
polypeptides
K erythropoietin
M G-CSF
R IFN-beta
X neuregulin
b oxytocin
tyrosine kinase inhibitors
O gefitinib
S imatinib
Z nintedanib

Fig. S14

Supplemental Figure Legends

Fig. S1 Type of response curves as a function of compound category. Four types of response curves were observed: no change, down-stroke, bimodal, upstroke. Histograms representing the percentage of each curve type in a compound category are shown. Separate analysis for pluripotent cells (panel A) and neurons (panel B). Significant enrichment of a response type in a compound category was estimated with hypergeometric probability distribution (* FDR < 0.05; ** FDR < 0.01; *** FDR < 0.001). FDR = false discovery rate (i.e. p-value corrected with Benjamin & Hochberg approach).

Fig. S2 (A) Values for lipid fraction (VFL), albumin concentration (P) and total protein concentration (P)* in rat and human plasma and in N2 (x) and BHK (y) medium. The total protein concentration was used for abiraterone IVIV-modelling only. (B-D) Values for plasma bound fraction (fb,pl); octanol:water partition coefficient (Kow) and molecular weight (MW). For each compound, the total concentration found to be toxic (or effective) in *in vivo* studies and the related free concentration are reported for different species (rat and human). Two parameters have been used: maximal concentration (Cmax) and average concentration (Cavg) calculated as the ratio of the area under the curve (AUC) on the dose interval (τ). These data have been used to calculate equivalent total (nominal) *in vitro* concentrations corresponding to *in vivo* toxic/effective concentrations for N2 medium (ECx) and BHK medium (ECy).

Fig. S3 and S4 Effect of well-known non-toxic compounds. Cells were exposed to compounds for 48h, neural (FLuc) and general (RLuc) promoter activities and DNA quantity (PI assay) were determined. Results are expressed as percent of control + SD. Mean control values (100%) is shown as dotted line; the SD of control values is shown as grey area. Data points that differ in a statistically significant manner from control values were determined by one-way repeated-measures ANOVA followed by Dunnett's post hoc test and are shown as filled circles. Data were obtained from 4-6 replicates.

Fig. S5 - S12 Effect of other compounds of ESNATS collection. Cells were exposed to compounds for 48h, neural (FLuc) and general (RLuc) promoter activities and DNA quantity (PI assay) were determined. Results are expressed as percent of control + SD. Mean control values (100%) are shown as dotted line; the SD of control values is shown as grey area. Data points that differ in a statistically significant manner from control values were determined by one-way repeated-measures ANOVA followed by Dunnett's post hoc test and are shown as filled circles. Data were obtained from 4-14 replicates.

Fig. S13 Graphical representation of the relevant concentrations listed in Fig.1 B (main text). Data in rats (blue), in clinical studies (green) and in our *in vitro* systems (ESC and neurons) are showed for each compound. The arrows indicate the concentration folds between the *in vitro* and the *in vivo* concentration ranges.

Fig. S14 A-C) A scatterplot of log10 transformed LOAEL. D) A principal component analysis (PCA) of compounds using orthogonal distances from the alert plot diagonals. For each compound orthogonal distances to the diagonals on alert plots (A-G) in the figure 6 were calculated, yielding 6 values for each compound. These were used as input for principal component analysis (PCA) where each dot represents a compound that was labeled according to its class in Table 2.

Supplemental Bibliography

- Eskenazi B, Chevrier J, Rauch SA, et al. (2013) In utero and childhood polybrominated diphenyl ether (PBDE) exposures and neurodevelopment in the CHAMACOS study. Environ Health Perspect 121(2):257-62 doi:10.1289/ehp.1205597
- Grandjean P, Herz KT (2015) Trace elements as paradigms of developmental neurotoxicants: Lead, methylmercury and arsenic. J Trace Elem Med Biol 31:130-134 doi:10.1016/j.jtemb.2014.07.023
- Grandjean P, Landrigan PJ (2006) Developmental neurotoxicity of industrial chemicals. Lancet 368(9553):2167-78 doi:10.1016/S0140-6736(06)69665-7
- Jentink J, Loane MA, Dolk H, et al. (2010) Valproic acid monotherapy in pregnancy and major congenital malformations. N Engl J Med 362(23):2185-93 doi:10.1056/NEJMoa0907328
- Kreyberg S, Torvik A, Bjorneboe A, Wiik-Larsen W, Jacobsen D (1992) Trimethyltin poisoning: report of a case with postmortem examination. Clin Neuropathol 11(5):256-9
- Krug AK, Kolde R, Gaspar JA, et al. (2013) Human embryonic stem cell-derived test systems for developmental neurotoxicity: a transcriptomics approach. Arch Toxicol 87(1):123-43 doi:10.1007/s00204-012-0967-3
- Morris E, Green D, Graudins A (2009) Neuroleptic malignant syndrome developing after acute overdose with olanzapine and chlorpromazine. J Med Toxicol 5(1):27-31
- http://www.ema.europa.eu/docs/en_GB/document_library/EPAR_-_Public_assessment_report/human/001016/WC500036361.pdf
- http://www.accessdata.fda.gov/drugsatfda_docs/label/2015/206995s000lbl.pdf
- Orr CF, Ahlskog JE (2009) Frequency, characteristics, and risk factors for amiodarone neurotoxicity. Arch Neurol 66(7):865-9 doi:10.1001/archneurol.2009.96
- Patejdl R, Markmann S, Benecke R, Wittstock M (2013) Severe acute motor neuropathy after treatment with triple tyrosine kinase inhibitor BIBF 1120 (Nintedanib). Clin Neurol Neurosurg 115(9):1851-2 doi:10.1016/j.clineuro.2013.01.011
- Reboli AC, Mandler HD (1992) Encephalopathy and psychoses associated with sulfadiazine in two patients with AIDS and CNS toxoplasmosis. Clin Infect Dis 15(3):556-7
- Rinne ML, Lee EQ, Wen PY (2012) Central nervous system complications of cancer therapy. J Support Oncol 10(4):133-41 doi:10.1016/j.suponc.2011.11.002
- Zimmer B, Pallocca G, Dreser N, et al. (2014) Profiling of drugs and environmental chemicals for functional impairment of neural crest migration in a novel stem cell-based test battery. Arch Toxicol 88(5):1109-26 doi:10.1007/s00204-014-1231-9
